# Role of Blood P-Tau Isoforms (181, 217, 231) in Predicting Conversion from MCI to Dementia Due to Alzheimer’s Disease: A Review and Meta-Analysis

**DOI:** 10.3390/ijms252312916

**Published:** 2024-11-30

**Authors:** Gemma Lombardi, Silvia Pancani, Riccardo Manca, Micaela Mitolo, Simone Baiardi, Federico Massa, Luigi Coppola, Monica Franzese, Emanuele Nicolai, Franca Rosa Guerini, Roberta Mancuso, Cristina Agliardi, Simone Agostini, Matteo Pardini, Gianni Virgili, Sandro Sorbi, Piero Parchi, Benedetta Nacmias, Annalena Venneri

**Affiliations:** 1 Department of Neurosciences, Psychology, Drug Research and Child Health (NEUROFARBA), University of Florence, Viale Pieraccini 6, 50139 Florence, Italy; gemma.lombardi@unifi.it (G.L.); gianni.vigili@unifi.it (G.V.); sandro.sorbi@unifi.it (S.S.); benedetta.nacmias@unifi.it (B.N.); 2 IRCCS Fondazione Don Carlo Gnocchi Onlus, Via di Scandicci 269, 50143 Florence, Italy; 3 Department of Medicine and Surgery, University of Parma, Via Volturno 39, 43125 Parma, Italy; riccardo.manca@unipr.it (R.M.); micaela.mitolo@unipr.it (M.M.); annalena.venneri@unipr.it (A.V.); 4 Department of Life Sciences, Brunel University London, Uxbridge UB8 3PH, UK; 5 IRCCS Istituto delle Scienze Neurologiche di Bologna, Via Altura 3, 40139 Bologna, Italy; piero.parchi@unibo.it; 6 Department of Biomedical and Neuromotor Sciences (DIBINEM), University of Bologna, Via Altura 1/8, 40139 Bologna, Italy; simone.baiardi6@unibo.it; 7 Department of Neuroscience, Rehabilitation, Ophthalmology, Genetics, Maternal and Child Health (DNOGMI), University of Genoa, Largo Paolo Daneo 3, 16132 Genoa, Italy; fedemassa88@gmail.com (F.M.); matteo.pardini@unige.it (M.P.); 8 IRCCS Ospedale Policlinico San Martino, Largo Rosanna Benzi 10, 16132 Genoa, Italy; 9 IRCCS SYNLAB SDN, Via Emanuele Gianturco 113, 80143 Naples, Italy; luigi.coppola@synlab.it (L.C.); monica.franzese@synlab.it (M.F.); emanuele.nicolai@synlab.it (E.N.); 10 IRCCS Fondazione Don Carlo Gnocchi Onlus, Via Capecelatro 66, 20148 Milan, Italy; fguerini@dongnocchi.it (F.R.G.); rmancuso@dongnocchi.it (R.M.); cagliardi@dongnocchi.it (C.A.); sagostini@dongnocchi.it (S.A.)

**Keywords:** Alzheimer’s disease, mild cognitive impairment, biomarkers, blood, plasma, p-tau

## Abstract

Blood-based biomarkers are minimally invasive tools to detect the pathological changes of Alzheimer’s Disease (AD). This meta-analysis aims to investigate the use of blood-derived p-tau isoforms (181, 217, 231) to predict conversion from mild cognitive impairment (MCI) to AD dementia (ADD). Studies involving MCI patients with data on blood p-tau isoforms at baseline and clinical diagnosis at follow-up (≥1 year) were included. Twelve studies on p-tau 181 (4340 MCI, conversion rate 20.6%), four on p-tau 217 (913 MCI, conversion rate 33.4%), and one on p-tau 231 (135 MCI, conversion rate 33%) were included. For p-tau 181, the pooled area under the receiver operating characteristic curve (AUC) was 0.73 (95% CI = 0.68–0.78), and for p-tau 217 was 0.85 (95% CI = 0.75–0.91). Plasma levels of p-tau 181 had good discriminatory power to identify MCI patients who will convert to ADD. Although only four studies on p-tau 217 have been included in the meta-analysis, in the last year the predictive power of p-tau 217 is emerging as superior to that of other isoforms. However, given the high heterogeneity detected in the p-tau 217 studies included in this meta-analysis, additional supportive evidence is needed. Insufficient results were available for p-tau 231. These findings support the prognostic utility of p-tau 181 and p-tau 217 measured in blood to predict progression to ADD in MCI and encourage its future implementation in clinical practice.

## 1. Introduction

Timely and accurate diagnosis of Alzheimer’s Disease (AD) in clinical practice is currently challenging, with misdiagnoses ranging from 20–25% in specialized centers [1,2] to higher percentages in primary care settings where routine cognitive screening is not performed and there is a lack of easily accessible, time- and cost-effective, and accurate diagnostic tools. As a consequence, there are frequent suboptimal, incorrect, or delayed interventions, as well as inaccurate communication about diagnosis and prognosis to patients and caregivers. In the last decades, the diagnosis of AD has undergone significant changes. The revolution in the diagnosis started in 2007, when the International Working Group for New Research Criteria for the Diagnosis of AD provided a new conceptual framework that moved AD from a clinical-pathological to a clinical-biological entity, proposing that AD could be detected in vivo using biomarkers before the appearance of dementia [3].

Later in 2018, the biomarker-based A-T-N (Amyloid/Tau/Neurodegeneration) research framework for AD was established indicating that biomarkers are necessary to support an in vivo diagnosis of AD, especially in the preclinical and prodromal phases of the disease when clinical symptoms are either absent or very subtle, but neuropathological changes are already ongoing and detectable [4]. To be in the “AD continuum” a positive amyloid marker identifiable by cerebrospinal fluid (CSF) as Aβ42 or Aβ42/Aβ40 ratio or, alternatively by Amyloid positron emission tomography (PET), is required.

Lastly, starting from recent advances in biomarker research and the consolidated habit to define disease biologically instead of using syndromic aspects, the research framework for AD was updated in April 2024 reporting objective criteria for AD diagnosis and staging also on the basis of blood-based biomarkers (BBMs) [5]. Blood Aβ42 and p-tau isoforms (p-tau 181, p-tau 217, p-tau 231) are recognized as Core 1 biomarkers in the revised criteria, as the CSF counterpart, and Amyloid PET. According to these criteria, an abnormal Core 1 biomarker result is sufficient to establish a diagnosis of AD, whereas later changes in Core 2 biomarkers (as non-phosphorylated mid-region tau fragments in biofluid and tau PET) are useful to provide prognostic information, and, if abnormal, increase confidence that AD is contributing to symptoms. Recent evidence suggests that combining blood p-tau 217 and Aβ42/Aβ40 levels could be useful for the early identification of symptomatic AD cases [6] and, potentially, for subject selection in primary prevention trials [7].

Among those for neurodegenerative diseases (NDDs), analysis of cerebrospinal fluid CSF and positron emission tomography (PET) measures are well-established markers with excellent diagnostic properties for AD. However, they are less useful outside very specialized clinics due to limited accessibility, invasiveness, and high costs, precluding the use of CSF- and PET-derived biomarkers in most primary and secondary care settings worldwide. For these reasons BBMs have emerged as promising tools to revolutionize the diagnostic and prognostic work-up of AD, as well as to improve the design of interventional trials. Indeed, BBM detection represents a less invasive and less costly approach than CSF or neuroimaging markers and is more feasible in primary care settings where most individuals with prodromal cognitive symptoms are assessed. Additionally, BBMs have been shown to differentiate Alzheimer’s Disease Dementia (ADD) from other dementias with accuracy levels similar to CSF and PET biomarkers. In contrast, standardized BBM methods to track individualized progression from a prodromal to a clinical stage of the disease, such as Mild Cognitive Impairment (MCI) to ADD, are still under definition. Despite the increased use of BBMs in research contexts to track the initial neuroanatomical changes of AD according to the Revised criteria for diagnosis and staging of AD, at present the clinical use of AD biomarkers is limited to the evaluation of symptomatic individuals, and their use is not recommended in cognitively unimpaired individuals [5]. This concept is also reinforced by the World Health Organization according to which individuals without established cognitive impairment do not represent a target population for BBMs at present (https://iris.who.int/bitstream/handle/10665/379286/9789240099067-eng.pdf?sequence=1, accessed on 17 November 2024).

Among the available BBMs, plasma Aβ42/40 ratio, p-tau isoforms, serum neurofilament light chain (NfL) and glial fibrillary acidic protein (GFAP) are the most advanced for diagnostic and prognostic purposes [8]. All these markers have specific characteristics and belong to different categories: Aβ42/40 ratio reflects “A” (Aβ proteinopathy, Core 1 biomarkers), p-tau isoforms 181, 217 and 231 reflect “T1” (phosphorylated and secreted AD tau, Core 1 biomarkers), NfL reflects “N” (injury, dysfunction, or degeneration of neuropil), GFAP reflects “I” (inflammation) [5].

P-tau shows higher specificity for AD, indeed an increase in this BBM has been observed only in AD and not in other tauopathies [9], levels being related to both the density of Aβ plaques and tau tangles, the neuropathological hallmarks of the disease [10]. It is worth noting that while p-tau isoforms are “T” biomarkers, several studies have demonstrated that they are highly correlated with Aβ pathology. In this regard, immuno and mass spectrometric assay (MSA) measures of p-tau 181, p-tau 217, and p-tau 231 have been shown to discriminate Aβ-positive from -negative individuals with high performance in many studies [10,11,12], and based on comparative analyses, there is evidence that p-tau biomarkers have a significantly higher correlation with Aβ PET than with tau PET [13].

Compared with CSF, blood-derived p-tau exhibits a lower concentration of p-tau species; however, blood p-tau change reflects tau changes in the CSF [14], and a significant correlation between CSF and blood levels of p-tau has been found [15]. Blood serum is also considered a viable medium for measuring p-tau concentration, as its accuracy has been demonstrated to be similar to that of plasma [16].

Beyond the specific protein species considered (i.e., p-tau 217, p-tau 181, and p-tau 231), plasma p-tau markers vary in their capability to predict AD according to the assay method and thresholds used [12,17,18].

Over a number of years, multiple laboratory methods have been developed to measure p-tau isoforms in the blood, ranging from manual methods, such as the enzyme-linked immunosorbent assay (ELISA), to fully automated ones, as the single-molecule array (Simoa) with HD-X Analyzer by Quanterix, ref. [19] that allows quantification down to sub-femtomolar concentrations (<1 pg/mL), or semi-automated methods such as Meso Scale Discovery (MSD) platforms that are more sensitive and require less sample volume than the conventional ELISA kit [20]. Three main assays have been developed to measure 3 different p-tau isoforms: assays for tau phosphorylated at the threonine amino acid 181 (p-tau 181), 217 (p-tau 217), or 231 (p-tau 231), using antibodies that are directed to the N-terminus or mid-domain of the protein, being these forms of tau present at much higher concentrations in blood due to proteolytic processing of tau from neurons into biofluids [21].

The thresholds for p-tau isoforms are currently under definition [6,9]. Such threshold points have been assessed mainly based on the detection of amyloid and/or tau pathology, and should also be tested widely in a clinical scenario to establish how the set cut-off points respond to given clinical conditions. In this regard, various methods can be used to determine single cut-offs to classify individuals as positive or negative, with 5–20% of individuals having a borderline level. For this reason and to reach an acceptable level of accuracy, the BBM Workgroup [22] proposed the approach of using two cut-offs, a higher and a lower one, to define three categories of results: positive, intermediate and negative. Because intermediate BBM test results do not provide information on amyloid status, the BBM Workgroup recommends to clarify the amyloid status performing further analyses (CSF/Amyloid PET) when an individual might be a potential candidate for anti-amyloid treatments. Alternatively, if amyloid status definition does not affect the short-term management of a patient, it is recommended that the BBM test is repeated at a later point (after 1 year).

Among the available BBMs, plasma p-tau has been shown to separate accurately ADD from other NDDs with high diagnostic accuracy [10,11,12,23,24], the largest relative increases in ADD are often observed for p-tau 217 [12,24,25], with an accuracy similar to that of the corresponding p-tau isoform measured in CSF [12,26,27,28].

In MCI, both plasma p-tau 181 and p-tau217 have shown to be promising in predicting accurately future cognitive decline and ADD conversion in the subsequent 2 to 6 years, again with an accuracy similar to that of the corresponding p-tau isoform measured in CSF [10,29].

Overall, blood p-tau 217 seems to have the largest fold-change between AD and non-AD disorders [12,24,25] and seems to be more related to AD conversion [30,31] compared with p-tau 181. Longitudinal increase of p-tau 217 is a marker of disease progression in preclinical and prodromal AD [28,32,33]. It is worth noting that a recent study found that the predictive power for AD conversion is significant for both p-tau 181 and 217 but lower than that for detection of cerebral amyloidosis [30]. Since in more recent years the diagnosis of AD has changed from a clinical-pathological to a biologically supported to an only biological definition, prominent recent studies have focused on the preclinical phase of the disease. In this respect, plasma p-tau 231 and p-tau217 have been recognized as state markers of amyloid-β pathology in preclinical AD [34].

Indeed, p-tau 217 shows an increase not only in the prodromal [35], but also in the preclinical stage of the disease, and can predict cerebral amyloid pathology assessed by amyloid PET [36]. It is worth noting that, according to Janelidze et al. [36], p-tau 217 shows the strongest association with amyloid pathology in MCI, but not in cognitive unimpaired individuals. Moreover, the fact that plasma p-tau 217 values start becoming abnormal before tau-PET, supports the idea of p-tau 217 as a biomarker able to track tau pathology at the earliest stages of AD development [37].

As for the temporal order of blood p-tau change, some studies suggest that p-tau 231 might be changing slightly earlier than the other p-tau markers, and might be altered before any deposition in the brain reaches the threshold for Aβ-PET positivity, and after Aβ42/40 begins to fall in CSF [11,15,34]. From a clinical point of view, Martinez-Dubarbie et al. [38] report that basal levels of p-tau231 correlate negatively with memory tests only in individuals with a sufficient amyloid load. This suggests that in the future p-tau 231 could be used to detect populations susceptible to AD in cognitive unimpaired individuals with detection before Aβ-PET positivity. Supplementary Table S1 shows shared and peculiar characteristics of p-tau isoforms.

To date, a meta-analysis by Li and Colleagues [39] has been conducted on the predictive value of blood p-tau 181 and 217 for detecting patients with MCI that will convert to ADD, with their results supporting the use of these BBMs as prognostic markers. This finding was obtained without assessing whether the diagnosis of ADD, when biologically defined, could impact the prediction results.

Thus, in an attempt to cover this gap, the primary objective of the present review and meta-analysis was to define the power of the blood p-tau isoforms of interest (181, 217, 231) for predicting conversion from MCI to ADD, with special attention to studies where the diagnosis had been biologically defined.

In line with this objective, the research question was as follows: “How predictive are blood-derived p-tau isoforms (181, 217, 231) of conversion from MCI to dementia due to AD?”

## 2. Methods

This meta-analysis was registered in the PROSPERO database (https://www.crd.york.ac.uk/prospero/display_record.php?ID=CRD42023472358, accessed on 20 August 2024) and conducted according to the Preferred Reporting Items for Systematic Review and Meta-Analysis (PRISMA) guidelines [40].

### 2.1. Search Strategy

We developed the search strategy in collaboration with the Library System Service of the University of Florence, a bibliographic research assistance service. Literature searches were conducted on the PubMed, Embase, and Cochrane Library databases.

**Figure 1:**
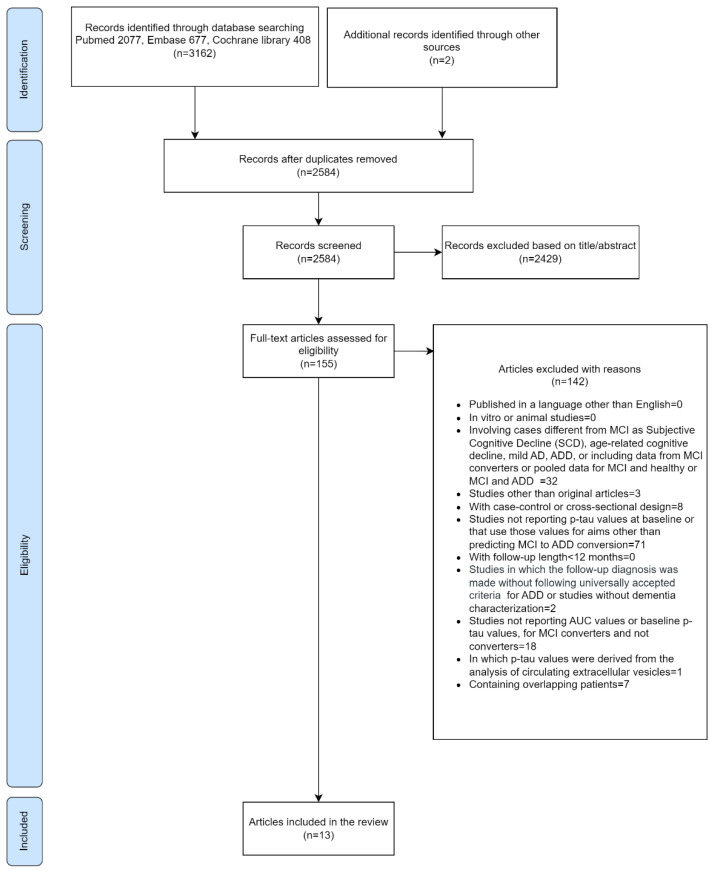
The flow chart of the literature selection.

We performed the most recent search for this review on 19 October 2023, identifying 2727 records. The search was then updated 1 year later, finding 478 new articles potentially eligible. Two additional records were identified through other sources. Overall, 3207 papers have been assessed for inclusion in this review (Figure 1).

The search string was created including terms that may be used to define the initial stage of the disease (MCI and equivalent terms as prodromal AD). Since MCI and Subjective Cognitive Decline (SCD) are often enrolled together in prospective studies, we decided to include in the search string also the term “preclinical AD” [41], for maintaining in a second step only data of MCI if available. In cases of referring to “early AD”, full texts were read to clarify whether the term was used for identifying “prodromal AD”. To include all studies of blood-derived p-tau, the terms plasma and serum were used as alternatives to blood. We restricted the search to the p-tau isoforms of interest (p-tau 181, p-tau 217, and p-tau 231). Finally, to capture studies with a prospective design we referred to a follow-up.

The full search strategy applied to each database is reported in Supplementary Tables S2–S4.

### 2.2. Study Eligibility

(a) We selected studies with a prospective longitudinal study design with a follow-up of 12 months or longer. In addition, historical cohorts such as disease registries (see ADNI) that included MCI cases and which recorded both p-tau values at baseline and the occurrence of any type of dementia, reversion, or other conditions during follow-up were selected. We included studies with extractable data; the hierarchy of data extraction was: sensitivity and specificity;
(b) area under the receiver operating characteristic curve (AUC) and standard error;
(c) mean/median p-tau values for both MCI converters and non-converters.

Referring to the “PICO model”, we defined inclusion and exclusion criteria as reported in Table 1.

### 2.3. Study Selection

Two authors (GL and SB) independently reviewed titles and abstracts of articles identified by the electronic database searches. If the study eligibility was in conflict, a third author (RM) solved any disagreement in the study selection. If there was still no agreement and eligibility could not be determined, the full text of the article was accessed to establish eligibility for inclusion on the basis of the criteria listed above. GL and SB independently further assessed the full manuscript against the inclusion criteria. Disagreement in the selection of full-text articles was solved by discussion. GL and SB also screened the references of selected articles and of the review of Li [39], and additional articles were not found through this strategy (i.e., there were no missed articles by the electronic database search), except one study that was retrieved from the review of Li. In the case of selection of more than one article from the same study authors or country, the absence of overlap in populations was assessed by using the reported recruitment periods or directly contacting the authors to define study eligibility. In the case of overlapping populations, the more recent study was retained, whereas in the case of multiple measurements of p-tau value with different assays in the same population, the more accurate measure was retained. The flow chart of the literature selection is reported in Figure 1.

### 2.4. Data Extraction

FM and SB extracted the following information from eligible studies: first author, year of publication, study country, name of the Clinic and that of study protocol, p-tau isoform tested, criteria used for MCI and ADD definition, follow-up length, details on the assay method used to measure p-tau in plasma or serum, number of MCI converted to ADD, MCI stable or converted to other dementia; percentage of females that participated in the study, age, ApoE status, education (years) and MMSE score at inclusion. When available, sensitivity/specificity, AUC and/or mean/median p-tau values were extracted for the meta-analysis. Where needed, authors were contacted for additional data.

### 2.5. Assessment of Methodological Quality

The Quality Assessment of Diagnostic Accuracy Studies-2 version (QUADAS-2) tool was used to assess the methodological quality of the included studies [51]. The review-specific QUADAS-2 scheme can be found in the Supplementary Material (Supplementary Table S5). A field is considered to exhibit a low risk of bias if all questions related to it are mostly answered with “yes”. However, if the answer to one or more signaling questions is “no”, the field is considered to exhibit a high risk of bias. Each paper was judged as having a ‘low’, ‘high’, or ‘unclear’ risk of bias for 4 domains: patient selection, index test, reference standard, flow and timing. We assessed concerns about applicability in 3 domains: patient characteristics and setting, index test, and reference standard. Clinical applicability was generally classified into three levels: “low”, “high” or “unclear”.

Studies were rated as low-quality in case of high or unclear risk of bias in at least one QUADAS-2 domain. Two authors (GL and RM) independently assessed the methodological quality of included studies and disagreement was solved by a third author (GV).

Considering that our review was focused on the biological definition of ADD, we judged the criteria for ADD a low risk of bias and low concern regarding applicability, only when the diagnosis was biomarker supported with amyloid markers measured in CSF assay or by PET (A+).

### 2.6. Statistical Analysis

Statistical analysis was conducted using Stata 18.0 (Stata Corp, College Station, TX, USA).

P-tau baseline values (average and standard deviation, SD) of converters and non-converters MCI were retrieved. If the study provided the median and interquartile range instead of mean and SD, the median was used as an estimate and mean and SD were estimated from the interquartile range (IQR) divided by 1.35. Because in the studies different techniques were applied to measure blood biomarkers, the standardized mean difference (SMD) was used as the principal measure of effect size so that the results could be combined. The SMD (Hedge adjusted g) were calculated for all the studies using differences in p-tau levels at baseline between MCI converters and non-converters divided by the standard deviations of differences pooled from the two groups. SMDs of 0.2, 0.5, and 0.8 are considered small, medium, and large, respectively [52], whereas a value of 0 is indicative of equivalence between p-tau levels in MCI converters and non-converters.

In addition, the area under the receiver operating characteristic curve (AUC) and its 95% confidence interval (CI) were recorded if available. When actual AUC and corresponding 95% CI were not available, they were computed from SMDs using established methodologies [53]. Summary AUC estimates were derived from a random-effects model utilizing restricted maximum likelihood (REML) estimation. The weight of each study in the meta-analysis was automatically calculated based on precision (1/variance).

An AUC of 0.5 suggests no discrimination ability of p-tau in detecting MCI converters from non-converters; up to 0.7 is considered low, 0.7 to 0.9 is considered moderate/acceptable, and more than 0.9 is considered high/excellent [54].

The heterogeneity across the included studies was quantified using the I^2^ statistic. A value of I^2^ of 0–25% indicates insignificant heterogeneity, 26–50% low heterogeneity, 51–75% moderate heterogeneity, and 76–100% high heterogeneity [55].

For inclusion in the meta-analysis, at least three articles were required to report data on the specific outcome (AUC or SMD). When appropriate, cross-validation was conducted according to the leave-one-out procedure to quantify the impact of potential outliers on the estimation of the overall effect size. A sensitivity analysis was conducted including only those studies using Aβ-markers (A+) to define ADD diagnosis, while a meta-regression was conducted to investigate the effect of follow-up duration and age.

### 2.7. Protocol Deviations

In the original protocol for extractable data, we intended to include only data necessary to construct a 2 × 2 table, that is, the number of true positive, false negative, true negative, and false positive cases. Due to the paucity of these data, we decided to include those studies reporting AUC or the mean p-tau values of MCI converters or non-converters to ADD. Moreover, in the original protocol, the ADD diagnosis was accepted only when a positive amyloid biomarker derived from a medium different from blood (i.e., Amyloid PET or Aβ under the cut-off value of normality in the CSF) supported the clinical diagnosis; however, at a later point, the decision was taken to include studies in which an ADD diagnosis had been made using clinical criteria because only a few studies were available that included biological confirmation of disease. Thus, we classified studies as A+ (where ADD diagnosis was performed with clinical criteria and assessing amyloid by CSF or PET) and A− (where ADD diagnosis was performed only with clinical criteria).

For the A+ studies including assessment of amyloid at baseline, eligibility was confirmed when amyloid status had been used at follow-up to confirm the ADD diagnosis (A_pos).

The statistical analysis was adapted based on data availability (lack of sensitivity and specificity).

## 3. Results

Twelve studies reported p-tau 181 levels for converters and non-converters MCI, for a total of 4340 participants (895 converted to ADD and 3445 not converted to ADD, total conversion rate 20.6%). Four studies reported p-tau 217 values, for a total of 913 participants (305 converted to ADD and 608 not converted to ADD, conversion rate 33.4%). One study reported p-tau 231 levels for a total of 135 participants (45 converted to AD and 90 not converted to ADD, conversion rate 33%).

### 3.1. P-Tau 181

Out of twelve studies, four enrolled clearly amnestic MCI [31,56,57,58], two enrolled both amnestic and non-amnestic MCI [59,60], one enrolled single- and multiple-domain amnestic MCI [61], while six studies did not specify the MCI type of participants involved [30,62,63,64,65,66]; however, the studies of Simren and Lehmann used the Petersen 1999 criteria for MCI selection, and Yuan 2024 is an ADNI study, thus, we can assume that these 3 studies include amnestic MCI. The mean age of participants ranged from 68 to 79.3 years for non-converters and 68 to 75.7 for converters to ADD. The mean follow-up duration ranged from 1 to 6 years. P-tau 181 values at baseline ranged from 1.5 to 46.3 pg/mL in the group of MCI who converted to ADD and from 0.9 to 23.2 pg/mL in the group of MCI who did not convert to ADD at follow-up. More details of included studies are provided in Table 2.

In all studies, values of p-tau 181 are expressed in pg/mL, reporting a wide range of values across studies measured with different laboratory methods. In nine studies, p-tau 181 was measured with Simoa [30,31,56,57,60,63,64,65,66], in one study with ELISA [61], in one study with MSD method [62], and in one study with the Prototype immunoassays on COBAS e 601 analyzer [59]. In only one study [60], p-tau was measured in serum instead of plasma.

**Figure 2:**
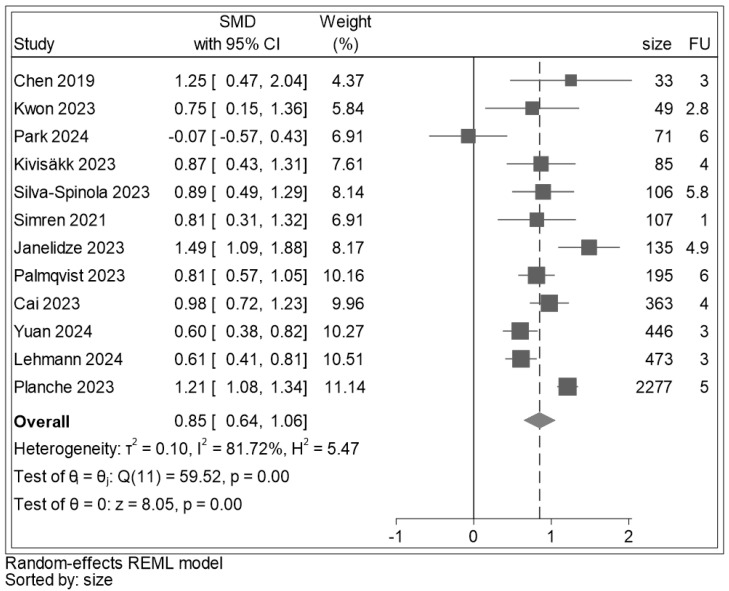
Forest plot for blood p-tau 181 of the SMD of the individual studies and their respective weight. A positive SMD indicates higher p-tau 181 values in participants who converted to ADD. The results of component studies are shown as squares centred on the point estimate of the result of each study. The horizontal line runs through the square to show its confidence interval. The overall estimate from the meta-analysis and its confidence interval are put at the bottom, represented as a diamond. The centre of the diamond represents the pooled point estimate, and its horizontal tips represent the confidence interval. The solid line is on 0 and corresponds to an equivalence between mean p-tau 181 value in MCI converters and non-converters to ADD; the dashed line is on the overall meta-analytic pooled-estimate on the SMD scale (that is 0.85). All studies [30,31,56,57,59,60,61,62,63,64,65] were beyond the 0 line except for Park 2024 [66]. Legend: size: sample size (number); FU: follow-up (years).

Baseline p-tau 181 data were extracted from all twelve studies (Figure 2). There was a significant difference between baseline p-tau 181 values in MCI converters and non-converters to ADD (SMD: 0.85, 95% CI: (0.64, 1.06), z = 8.05, *p* < 0.001), indicating higher p-tau 181 values in those converting to ADD. The heterogeneity index I^2^ was 81.72%.

**Figure 3:**
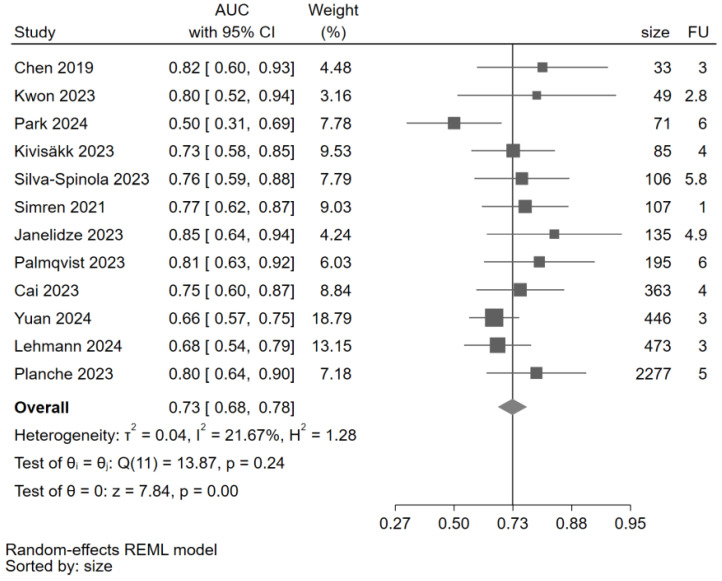
Forest plot for AUC of p-tau 181 estimated from the individual studies and their respective weight. Legend: AUC: area under the receiver operating characteristic curve; CI: confidence interval; size: sample size (number); FU: follow-up (years). The solid line corresponds to an AUC of 0.73, a value considered acceptable for the ability of p-tau 181 to discriminate between MCI converters and non-converters. All studies [30,31,56,57,59,60,61,62,63,64,65] were beyond this line except for Park 2024 [66].

AUC values were extracted or estimated for all twelve included studies (Figure 3). Across studies, p-tau 181 showed a discrimination ranging from 0.68 to 0.78. The overall discrimination estimate was 0.73 (95% CI: (0.68–0.78), z = 7.84, *p* < 0.001), thus, in an acceptable range. Heterogeneity detected across studies was 21.67%. The leave-one-out meta-analysis showed an overall effect size of 0.74 computed excluding the study of Park [66] (Table 3).

**Figure 4:**
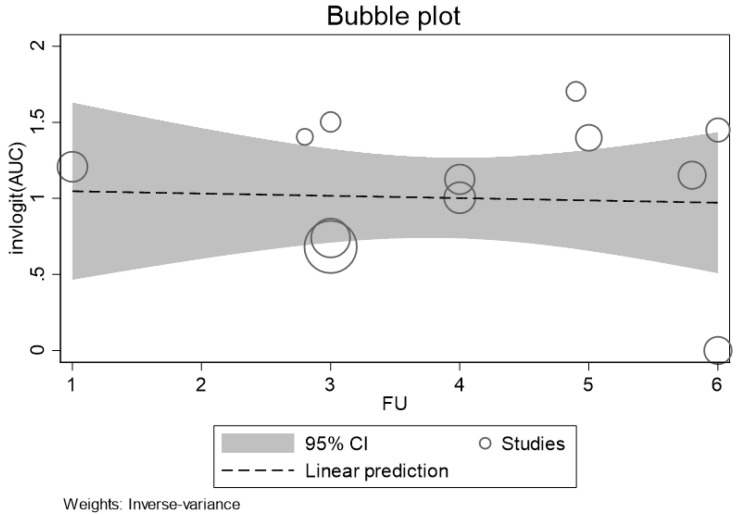
Bubble plot showing the logitAUC (effect size) of p-tau 181 estimated for the individual studies plotted (circle) against follow-up duration. Legend: FU: follow-up.

The bubble plot graphically examined the estimated relationship between AUC of p-tau 181 (after logit transformation) and follow-up duration. The size of the points is proportional to the weight the studies received in the analysis. The meta-regression results displayed in Figure 4 show the absence of a linear relation between increasing follow-up duration and AUC.

**Figure 5:**
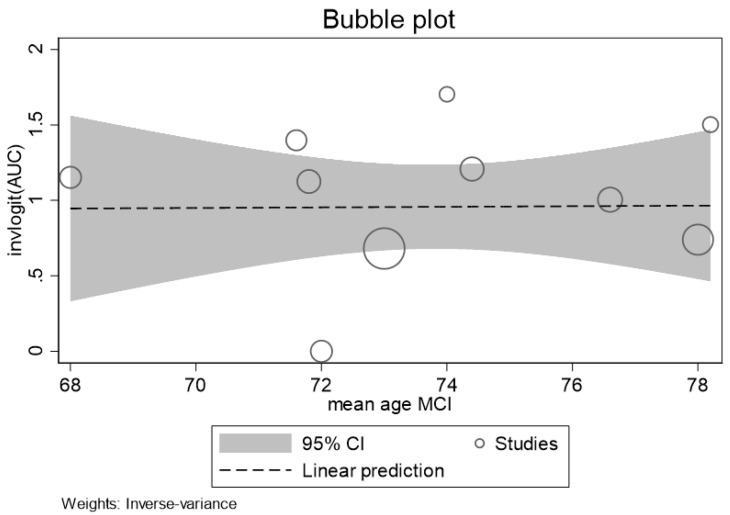
Bubble plot showing the logitAUC (effect size) of p-tau 181 estimated for the individual studies (circle) plotted against participants’ age (years).

Similarly, no significant linear relation between increasing participants’ age and discrimination ability of p-tau 181 was observed (Figure 5).

**Figure 6:**
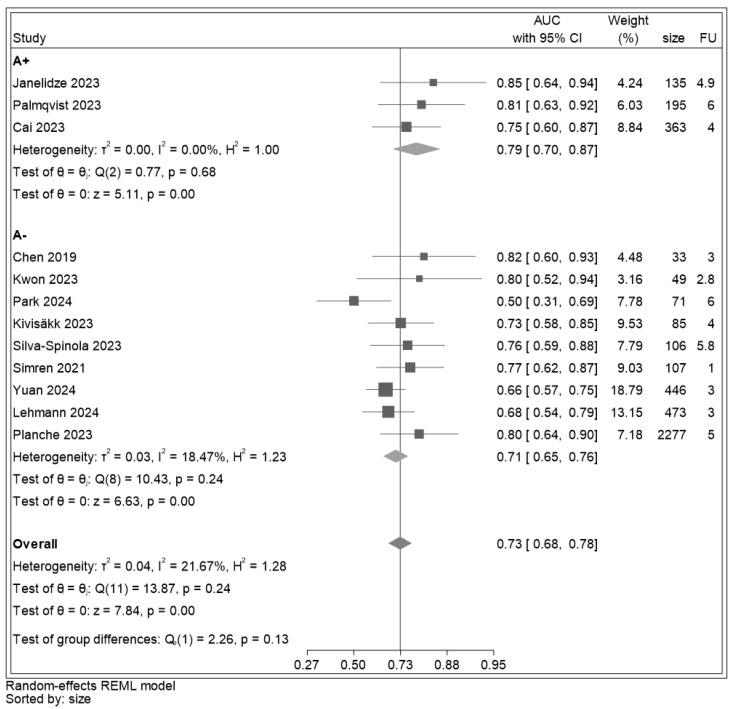
Forest plot for AUC of p-tau 181 estimated from A+ studies (biologically confirmed ADD diagnosis) and from A− studies (only clinical ADD diagnosis). Legend: size: sample size (number). The solid vertical line set on the pooled AUC, 0.73. The AUC in the A+ studies (0.79) was superior to the AUC in the A− studies (0.71), but the difference was not statistically significant [30,31,56,57,59,60,61,62,63,64,65,66].

The results of the sensitivity analysis, including studies reporting data separately for A+ and A−, show that the discrimination estimate of p-tau 181 for MCI with an additional amyloid marker tested at baseline (in CSF or brain with PET) and used to confirm the diagnosis of ADD at follow-up, was 0.79 (95% CI: (0.70–0.87), z = 5.11, *p* < 0.001) while in MCI without amyloid marker availability, it was 0.71 (95% CI: (0.65–0.76), z = 6.63, *p* < 0.001) (Figure 6). The difference in AUC in A+ vs. A− studies was not significant (*p* = 0.13).

### 3.2. P-Tau 217

Out of four studies, two enrolled amnestic MCI [31,58], while one enrolled both amnestic and non-amnestic MCI [59]; lastly, Lehmann used the Petersen 1999 criteria, thus included amnestic MCI, but he did not report cohort description in this study [30]. Three studies reported the mean age of participants [30,47,49], 74.0, 71.5, and 77.7 years respectively, whereas one study did not report this information. The mean follow-up duration ranged from 3 to 6 years. P-tau 217 values at baseline were recorded in three studies (Refs. [30,31,58], mean values 0.62 pg/mL and 3.81 for MCI converted to ADD and 0.25 pg/mL and 2.93 for MCI not converted to ADD). More details of the included studies are shown in Table 2.

P-tau 217 was measured in one study with MSA [31], in one with MSD [58], in one with a prototype method with the COBAS e 601 analyzer [59], and in one study with the Simoa ALZpath using monoclonal p-tau specific antibodies commercially available [30].

**Figure 7:**
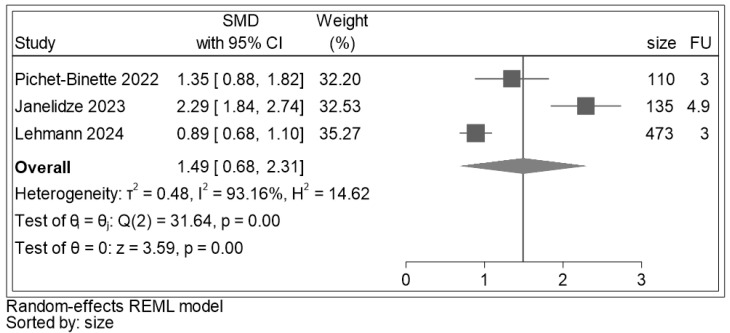
Forest plot for blood p-tau 217 of the SMD of the individual studies and their respective weight. A positive SMD indicates higher p-tau 217 values in participants who converted to ADD. Legend: size: sample size (number); FU: follow-up (years); the solid vertical line set on overall SMD results (1.49), [30,31,58].

Baseline p-tau 217 data were extracted from three out of four studies (Figure 7). There was a significant difference between baseline p-tau 217 values in MCI converters and non-converters to ADD (SMD: 1.49, 95% CI: (0.68,2.31), z = 3.59, *p* < 0.001), indicating higher p-tau 217 values in those converting to ADD. The heterogeneity index I^2^ was 93.16%.

**Figure 8:**
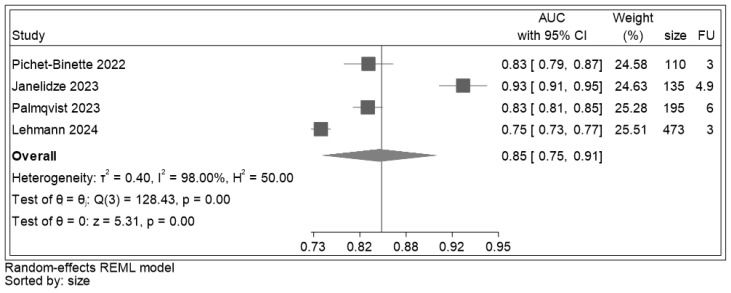
Forest plot for AUC of p-tau 217 estimated from the individual studies and their respective weight. Legend: AUC: area under the receiver operating characteristic curve; CI: confidence interval; size: sample size (number); FU: follow-up (years). The solid vertical line is set on the pooled AUC for p-tau 217 (0.85), [30,31,58,59].

AUC values were extracted or estimated for all four included studies (Figure 8). Across studies, p-tau 217 showed a discrimination ranging from 0.75 to 0.91, thus from an acceptable to an excellent range of AUC. The overall discrimination estimate was 0.85 (95% CI: (0.75–0.91), z = 5.31, *p* < 0.001). Heterogeneity detected across studies was high (I^2^ = 98%).

### 3.3. P-Tau 231

The only study retrieved from the study search and selection process included amnestic MCI followed over a mean time of 4.9 years (SD: 2.1) [46]. The mean p-tau 231 value recorded at baseline was 16.8 pg/mL, 22.0 pg/mL, and 26.9 pg/mL in non-converters A_neg, non-converters A_pos, and converters to ADD, respectively. Details are reported in Table 2. The association of plasma p-tau 231 with future conversion to ADD was good (AUC: 0.78, 95% CI: (0.70–0.86)).

### 3.4. Quality of Included Studies

Only the study of Planche [60] was considered at low risk of bias for the participants’ inclusion. In contrast, only the study of Silva-Spinola [63] was considered at high risk of bias because it did not avoid inappropriate exclusion. All the other studies were considered at unclear risk of bias since there were missing details about patients’ enrollment criteria.

For the index test, only two studies were at low risk of bias. In these studies, a pre-specified cut-off was defined: in the study of Kivisakk [62], an optimal p-tau181 threshold to differentiate ADD from healthy control was calculated using the Youden index, and this threshold was applied to the MCI sample, whereas in the study of Planche [60], tertiles were used to estimate the cut-off because no cut-off values for BBMs are available to date. All other studies were classified as unclear risk for missing details on the threshold used. For the reference standard, in 4 studies the ADD diagnosis was biomarker-supported (A+) [31,56,58,59]; however, only the study of Pichet-Binette [58] was judged at low risk of bias since only this study reported blindness to plasma biomarker results. All other studies were classified at unclear risk for this domain. In 9 studies [30,31,56,57,61,62,64,65,66] the flow and time domains were judged at low risk of bias, the other 4 studies [58,59,60,63] received a high risk of bias for not reporting reasons for withdrawals. In the study of Silva-Spinola [63], only 4 cases were excluded from the analysis because these did not convert to ADD.

## 4. Discussion

The aim of this review and meta-analysis was to define the predictive role of the blood p-tau isoforms of interest (p-tau 181, p-tau 217, and p-tau 231) as predictors of conversion from MCI to ADD.

Additionally, the impact of using different criteria for the ADD diagnosis (only clinical or even biomarker-supported with already validated biomarkers) on this outcome was assessed. The review was focused on p-tau isoforms, the most promising BBM characterized by peculiar characteristics as having a significantly high correlation with Amyloid PET, as well as with established CSF markers, and their level of prediction of conversion to ADD in MCI (see Supplementary Table S1). The identification of prodromal AD is of paramount importance in order to properly select individuals eligible for anti-amyloid treatments with currently FDA-approved drugs. To this end, since amyloid deposition is common in cognitively impaired patients suffering from a brain disorder different from AD [67], with current data revealing 29% amyloid positivity in patients with non-AD brain disorders [68], p-tau isoforms are considered very useful for increasing the specificity for the identification of AD pathology.

Thirteen studies have been included in the meta-analysis (12/13 with data on p-tau 181 and 4/13 with data on p-tau 217). In all studies, values of p-tau 181 were expressed in pg/mL, with a wide range of values depending on the laboratory method used and the fluid where the marker had been measured. Moreover, a different dilution (2 versus 4-fold) can be applied to the samples in the pre-analytical phase, influencing the lower limit of detection. The majority of studies reported measurements obtained using Simoa (9 studies). However, values are not comparable among studies, not even when analyzed with the same method, because of the different use of assays, e.g., a commercial one or an in-house returning values with a 10x difference. The study of Janelidze [31] used the prototype Simoa assay ADx Neurosciences. Additionally, to our knowledge, p-tau values obtained with different methods are not convertible among them.

In comparison with the meta-analysis of Li 2023 [39], we included only 13 studies instead of 16, excluding those with a high risk of overlap in population, mainly based on ADNI and Biofinder participants. Two ADNI studies (Cai and Yang) were included in our meta-analysis since different methods were used to perform ADD diagnosis. In detail: the study of Janelidze [10] was excluded because of overlap with Palmqvist (Ref. [59] Biofinder study), the study of Cullen [69] was excluded because of an overlap with Palmqvist (Biofinfer study) and Cai (Ref. [56] ADNI study), the study of Karikari [70] was excluded for overlap with Cai [56], as well as the study of Shen [71] and Therrieult [72], with data from the latter study also not being extractable. The study of Palmqvist [29] was excluded due to overlap with Cai [56] (ADNI cohort) and with Palmqvist [59] (Biofinder cohort), the study of Kivisakk [73] was excluded because of an overlap with that of the same Authors (Ref. [62] MADRC study), the study of Xiao [74] because the predictive value of blood p-tau was assessed in combination with blood Aβ, and the study of Lehmann [75] was excluded because it was not clear whether risk of conversion had been estimated for ADD or for overall dementia. All studies included in the article by Li [39] were retrieved by the search string of this review, except that of Shen [71]. As for their results, the pooled ratio of means of p-tau 181 and 217 was higher in MCI converters than in MCI non-converters. These data were comparable with ours using SMD. In the study by Li [39], the overall discrimination estimate was greater for p-tau 217, 0.93 (95% CI: (0.90–0.95)) than for p-tau 181, 0.86 (95% CI: (0.83–0.89)) and, in the same way, in our study, the overall discrimination estimate was greater for p-tau 217, 0.85 (95% CI: (0.75–0.91)) than for p-tau 181, 0.73 (95% CI: (0.68–0.78)). For the AUC obtained for p-tau 181, the leave-one-out procedure revealed that excluding the study of Park [66] from the meta-analysis resulted in the highest AUC (0.74). This is because in the study of Park, the mean values of p-tau 181 are not significantly different between MCI converters and MCI non-converters, as shown in Table 2. In our review, the diagnosis of ADD was biomarker supported in 3 out of 4 of the p-tau 217 studies, whereas it was biomarker supported in 3 out of 12 of the p-tau 181 studies. Thus, a sensitivity analysis was conducted on the A+ studies only for p-tau 181, with the results showing that the discrimination estimate of p-tau 181 for MCI in the A+ studies was superior but not significantly higher than that obtained in the A− studies (AUC 0.79 vs. 0.71).

Although including different original articles, the 2 reviews highlight the predictive value of p-tau isoforms with a similar trend, supporting p-tau 217 as the most promising isoform.

In the meta-regression analysis, similarly to Li et al. [39], we found that the length of follow-up and the age of participants did not change the predictive power of p-tau 181, suggesting that this marker exerts its power in a wide range of years of follow-up (from 1 to 6 years), without any significant difference in the range of age assessed (68–79).

Finally, heterogeneity assessed in our meta-analysis was low for the studies of p-tau 181 included in the AUC analysis (I^2^ = 21.67), whereas in the other analyses it was high, especially for p-tau 217. Because of the high level of heterogeneity detected in the p-tau 217 studies included in this meta-analysis, additional evidence is needed to confirm the superior predictive power of p-tau 217 compared with p-tau 181.

It is well known that plasma p-tau is highly correlated with CSF p-tau values [15]. Similar results were obtained in a meta-analysis including 23 studies that assessed the role of CSF p-tau in predicting conversion from MCI to ADD [76], where SMD was highly significant (*p* < 0.0001) for the comparisons between stable MCI versus MCI converted to ADD (−1.03 [CI −1.47, −0.59]). Moreover, the CSF Aβ(1–42)/p-tau ratio differentiated converters from non-converters MCI robustly. This result was confirmed in CSF by the meta-analysis of Salvado [77], where an AUC value of 0.92 was estimated in the Biofinder 1 cohort and of 0.87 in the Biofinder 2 cohort, including also age, sex, APOE-ε4 status in the model. These results suggest a possible advantage of using this combination of biomarkers also in the blood.

Overall, the results from this study encourage the adoption of blood-derived p-tau isoforms for clinical use in two main settings in the near future, as suggested by Scholl et al. [78]:

(1) in primary care, to reduce waiting times and costs of diagnosis to improve diagnostic accuracy and to streamline referrals;
(2) in specialized centers as a first evaluation step for cognitive disturbances to select patients for subsequent more complex evaluation, with the purpose of streamlining the diagnostic process and for patient selection, stratification, and monitoring of therapeutic effects in clinical trials.

However, since the risk of potential overdiagnosis exists, according to Jack et al. [5], and recent suggestions from the World Health Organization (https://www.who.int/publications/i/item/9789240099067, accessed on 17 November 2024), the target population for BBMs is represented by early symptomatic cases.

Thus, potential population screening in the future still represents the most challenging use [79] and is not recommended at this time.

Moreover, since the same biomarkers can play different roles, especially when different thresholds are used (e.g., lower thresholds to screen patients for additional evaluation such as PET or CSF assays, and higher thresholds to confirm the presence of the disease), different cut-offs need to be determined for these purposes.

There are, however, some concerns about the use of p-tau in clinical settings: an important risk of applying only p-tau markers is the underdiagnosis of non-AD pathologies and other conditions potentially contributing to cognitive disorders in patients with negative results [80]; some factors affect biomarker concentrations such as age, sex [81], and several comorbidities including chronic kidney disease, hypertension, stroke, and myocardial infarction [18], and their impact on the adopted threshold should be established. In this regard, Bouteloup et al. [82] demonstrated that the variance in AD BBM concentrations was mainly explained by age, with minor contributions from cognition, brain atrophy, and genetics, differently from CSF measures, challenging, therefore, the use of BBMs as isolated stand-alone biomarkers of AD.

Finally, in order to overcome interference of non-AD copathology on BBM interpretation, VCAM1/ICAM1 and alpha-synuclein are emerging in research as new BBMs suitable for the identification and characterization of non-AD copathology such as vascular pathology and alpha-synucleinopathy, respectively [83].

Limitations and strengths in our meta-analysis should be acknowledged. Since several studies did not report detailed information on the characteristics of the MCI sample, e.g., whether amnestic single or multiple domains or whether not amnestic, we decided not to perform a sensitivity analysis for MCI subtypes. In addition, data extraction raised some concerns: for a number of studies, data could not be directly extracted from the articles to construct a 2 × 2 table nor obtained from the authors to calculate sensibility and specificity values. As a consequence, we performed a meta-analysis limited to the SMD and the AUC for p-tau 181 and 217. Due to the small number of studies included, meta-regression analysis was performed only for p-tau 181. The predictive value of p-tau isoforms might, therefore, have been underestimated due to insufficient research; on the contrary, the use of repetitive measures in the same samples (as in the study of Janelidze et al. [31]) might have led to overestimation of findings. Moreover, several of the included studies have been carried out with individuals who are part of historical cohorts collected in specialized centers and therefore not representative of the general population of patients seen in regular memory clinics. Unfortunately, only one study analyzing blood p-tau 231 and only 4 studies analyzing blood p-tau 217 were retrieved by our search, making it difficult to establish the predictive power of these isoforms. Only a few of the studies included in this review and meta-analysis [30,31,59] compared the AUC of p-tau 217 and p-tau 181 directly in the same cohort, reporting a higher value for p-tau 217 in the ability to discriminate between MCI converters and non-converters in 2 out of 3 studies (0.746 vs. 0.677 and 0.932 vs. 0.846, [30,31]). Lastly, heterogeneity also is challenging to interpret the findings of p-tau 217; thus, more studies are needed to validate our results. However, for the findings of p-tau 181, compared with the meta-analysis of Li [39], our work is updated and enriched by a sensitivity analysis focused on studies where the ADD diagnosis had been biomarker-supported (A+) and did not include overlapping populations.

## 5. Conclusions

This meta-analysis supports the predictive role of blood p-tau 181 for conversion from MCI to ADD and encourages its future implementation in clinical practice. Of note, the predictive effects of p-tau 181 appear to be not sensitive to participants’ age and length of follow-up for the range of years examined in the present study (age 68–79, 1 to 6 years). P-tau 217 was identified as the most promising isoform in terms of prediction; however, since only 4 studies with high heterogeneity were included in the meta-nalysis, additional supporting evidence is needed to confirm this finding. Insufficient results are available from our review about p-tau 231.

Overall, further validation studies are needed before adoption of blood p-tau isoforms in the routine diagnostic work-up of people experiencing cognitive decline, in order to validate cut-offs, harmonize inter-laboratory methods, and define the impact of confounding factors.

## Figures and Tables

**Table 1:** Inclusion and exclusion criteria.

Inclusion Criteria	Exclusion Criteria
Participants: we included studies conducted on humans, without age limits, involving MCI of all subtypes (amnestic-/non-amnestic, single or multiple domains) in accordance with the clinical definition of MCI (or terms considered equivalent as prodromal AD, cognitively impaired no dementia, minor neurocognitive disorder), according to the criteria of Albert et al. [42], Petersen et al. [43,44], Winblad et al. [45], and to definition used in the Diagnostic and Statistical Manual of Mental Disorders (V edition and previous) [46], or based on a CDR score definition of 0.5 [47]; Index test: we have included studies that measured p-tau isoforms of interest for the review and meta-analysis (181, 217, and 231) using any laboratory methods (from manual as ELISA to automatic as Simoa HD-X) in plasma and serum. MCI individuals with higher levels in 1 of the 3 isoforms of blood p-tau were considered at greater risk of conversion to ADD in comparison with MCI individuals with lower levels; Target condition/reference standard: we selected studies where the target condition was ADD diagnosis according to universally accepted clinical criteria (NINCDS-ADRDA criteria of McKhann et al. [48], NIA-AA criteria of McKhann et al. [49], IWG criteria of Dubois et al. [3], IWG-2 criteria of Dubois et al. [50]) with and without biological confirmation of the disease [4], defined as the identification of a positive amyloid marker detected by CSF or PET (A_pos).	Published in a language other than English (reason for exclusion number 1); Involving animals or in vitro studies (reason for exclusion number 2); Involving cases different from MCI as SCD, age-related cognitive decline, mild AD, ADD, or including only data of MCI converters or pooled data for MCI and healthy/SCD/ADD (reason for exclusion number 3); Other than original articles (reason for exclusion number 4); With a case-control or a cross-sectional design (reason for exclusion number 5); Not reporting p-tau values at baseline or using those values for aims other than predicting MCI to ADD conversion (reason for exclusion number 6); With a follow-up length of less than 12 months (reason for exclusion number 7); In which the follow-up diagnosis was made without following the universally accepted criteria for ADD, or studies without dementia characterization (reason for exclusion number 8); Not reporting sensitivity/specificity, AUC values or baseline p-tau values for MCI converters and non-converters (reason for exclusion number 9); In which p-tau values were derived from the analysis of circulating extracellular vesicles (reason for exclusion number 10); Containing overlapping participants (cases belonging to the same database such as for example ADNI) (reason for exclusion number 11)

**Table 2:** Characteristics of included studies.

Author, Year (Study Country) Clinic (Name of Study Protocol)	P-Tau Isoform	MCI Criteria	ADD Criteria (Underlined Whether A+ Applied)	FU (Mean, SD) Years	Assay Method (Serum/ Plasma)	MCI Type	MCI Converted to ADD (n)	MCI Stable + MCI Converted to Other D (n + n = tot)	Mean ± SD or Median (IQR) P-Tau (pg/mL) Values MCI Converters vs. MCI Non-Converters	Age (Mean, SD or Median IQR)	ApoE4 Carriers (%)	MMSE Score (Mean, SD or Median IQR)
Janelidze, 2023 (Sweden) Memory Clinic, Skåne University Hospital, Malmö [31]	p-tau 181^ADx^ p-tau 217^WashU^ p-tau 231^UGOT^	Petersen 2004 [44]	DSM-IIIR for dementia and probable ADD (NINCDS-ADRDA) and A+ (abnormal CSF Aβ42/40 ratio) (A+ measured baseline)	4.9, 2.1	Simoa HD-X (plasma) ADxNeurosciences (prototype) MSA (plasma) Simoa HD-X (plasma)	Amnestic	45	90 (not specified separated numbers)	Median value reported according to Aβ status	74.0 (66.0–79.0)	55.6	28.0 (26.0–29.0)
Cai, 2023 (USA, Canada) (ADNI) [56]	p-tau 181	MMSE 24–30, CDR 0 or 0.5 with a memory box score ≥ 0.5. Objective evidence of memory impairment	Probable ADD (NINCDS-ADRDA), A+ (Aβ42 < 192 pg/mL) MMSE 20–26, CDR of 1 or higher and A+ (abnormal CSF Aβ42) (A+ measured baseline)	4	Simoa HD-1 (plasma) in-house	Amnestic	85	264 + 14 = 278	26.4 ± 15.2 vs. 16.0 ± 8.8	71.8, 7.3	47.8	28.0 (1.8)
Palmqvist, 2023 (Sweden) Memory Clinic, Skåne University Hospital, Malmö (BioFINDER-1) [59]	p-tau 181^Abs^ p-tau 217^N-terminal-Roche^	DSM-5 and evidence of domain z-scores of ≤−1.5 in at least one cognitive domain	Major neurocognitive disorder due to AD (DSM-5) and anormal Aβ42/40 ratio or ^18^F-flutemetamol PET (A+ measured baseline)	6	Prototype immunoassays on COBAS e 601 analyzer (plasma)	Amnestic and non-amnestic	99	30 + 66 = 96	mean value available only according to Aβ status (not to conversion)	n/a	n/a	n/a
Pichet Binette, 2022 (Sweden) (NCT01028053, 2009–2014 trial, promotor GE Healthcare, USA) [58]	p-tau 217	Petersen 2004 [44]	probable AD (NINCDS-ADRDA) and A+ (^18^F-flutemetamol PET) (A+ measured baseline)	3	MSD (plasma)	Amnestic	26	71 + 13 = 84	0.62 ± 0.36 vs. 0.25 ± 0.24	72.28, 8.2	25.0	n/a
Planche 2023 (French) 26 French Memory Clinics (Memento study) [60]	p-tau 181	CDR = 0.5	DSM-IV criteria for dementia, NIA-AA criteria for ADD	5	Simoa HD-X (serum) Quanterix	Amnestic and non-amnestic	257	1957 + 63 = 2020	1.5 (1.0; 2.1) vs. 0.9 (0.6; 1.2)	71.6(65.5–77.1)	n/a	n/a
Kwon, 2023 (Republic of Korea) 9 Hospitals (KBASE-V study) [57]	p-tau181	Modified Petersen 2004 [44] and Albert (NIA-AA) 2011 [42]	McKhann (NIA-AA) 2011	2.8	Simoa HD-X (plasma) Quanterix	Amnestic	16	33 (not specified whether stable or not)	3.6 ± 2.9 vs. 2.1 ± 1.3	n/a	n/a	n/a
Chen, 2019 (Taiwan) memory clinic at Taipei Veterans General Hospital [61]	p-tau 181	Albert (NIA-AA) 2011 [42]	McKhann (NIA-AA) 2011	3	ELISA (plasma)	Amnestic (single or multiple domains)	10	23 (all stable)	25.8 ± 2.4 vs. 23.2 ± 1.9 (values reported as mean and standard errors)	79.3 (76.6–82.7) (non-converters to ADD); 75.8 (73.3–82.1) (converters to ADD)	36.4	28.0 (27.0–28.0) (non-converters to ADD); 25.0 (22.7–26.2) (converters to ADD)
Kivisäkk, 2023 (USA) Massachusetts (MADRCLC study) [62]	p-tau 181	Albert (NIA-AA) 2011 [42]	McKhann (NIA-AA) 2011	4	MSD (plasma)	n/a	47	38 (all stable)	2.9 (1.4) vs. 1.73 (1.13)	76.6, 8)	n/a	27, 2.9
Silva-Spinola, 2023 (Portugal) Neurology Department of Coimbra University Hospital [63]	p-tau 181	Albert (NIA-AA) 2011 [42]	McKhann (NIA-AA) 2011	5.8, 3.4	Simoa SR-X (plasma) Quanterix	n/a	60	46 (all stable)	2.0 (1.5–3.1) vs. 1.1 (0.7–1.6)	68.0 (60.0–73.0)	42	27.6, 2.2
Simrén, 2021 (Europe) 6 countries across Europe, Italy included (AddNeuroMed study) [64]	p-tau 181	Petersen 1999 [43]	DSM-IV and probable AD (NINCDS-ADRDA)	1.0, 0.1	Simoa HD-1 (plasma) in-house	Probably amnestic	19	88 (all stable)	17.13 (6.19) vs. 12.26 (5.89)	74.5, 5.9	36.4	27.2, 1.8
Park, 2014 (Republic of Korea) (KLOSCAD study) [66]	p-tau 181	Petersen 2004 [44]	DSM-IV and NINCDS-ADRDA	6	Simoa (plasma) Advantage V2.1 assay	n/a	21	50 (stable)	19.2 (14.2–39.4) vs. 20.2 (11.5–27.2)	74 (71–78) in converters vs. 71 (65–75) in non-converters	33 in converters vs. 22 in non-converters	21 (19–26) in converters vs. 24 (21–26) in non-converters
Yuan 2024 (Canada and USA) (ADNI) [65]	p-tau 181	As in Cai, 2023 [56]	NINCDS-ADRDA criteria	2.9	Simoa (plasma) in house assay Univ. Gothenburg (Sweden)	amnestic	101	345 stable	24.51 (14.58) vs. 17.26 (11.11)	72.7, 6.8	66 in converters vs.41 in non-converters	n/a
Lehmann 2024 (France) (Baltazar study) [30]	p-tau 181 p-tau 217	Petersen 1999 [43]	CDR progression	3	Simoa (plasma) Quanterix HD-X Advantage V1 kit Simoa ALZpath (plasma)	Probably amnestic	135	338	3.81 (1.54) vs. 2.93 (1.39) 0.69 (0.37) vs. 0.41 (0.29)	77.7 (5.5)	39	26.4 (2.5)

**Table 3:** Results from the leave-one-out procedure.

Omitted Study	AUC	95% CI	
Chen 2019 [61]	0.724	0.671	0.771
Kwon 2023 [57]	0.727	0.674	0.774
Park 2024 [66]	0.740	0.693	0.783
Kivisäkk 2023 [62]	0.732	0.674	0.783
Silva-Spinola 2023 [63]	0.728	0.672	0.778
Simrén 2021 [64]	0.726	0.670	0.776
Janelidze 2023 [31]	0.722	0.670	0.768
Palmqvist 2023 [59]	0.723	0.670	0.770
Cai 2023 [56]	0.729	0.672	0.779
Yuan 2024 [65]	0.743	0.688	0.791
Lehmann 2024 [30]	0.739	0.681	0.790
Planche 2023 [60]	0.722	0.669	0.770
Overall	0.729	0.678	0.775

## Data Availability

The datasets used and analyzed during the current study are available from the corresponding author upon reasonable request. Data are also extractable directly or calculable from original articles included in the review.

## Supplementary Materials

The following supporting information can be downloaded at: https://www.mdpi.com/article/10.3390/ijms252312916/s1. References [84,85,86] are cited in Supplementary Materials

## Abbreviations

A_pos	Amyloid positivity assessed by Amyloid PET or cerebrospinal fluid analysis
A_neg	Amyloid negativity assessed by Amyloid PET or cerebrospinal fluid analysis
A+	Study where ADD diagnosis was performed with clinical criteria and assessing amyloid by CSF or PET
A−	Study where ADD diagnosis was performed with clinical criteria only
Aβ	Beta-amyloid
AD	Alzheimer’s Disease
ADD	Alzheimer’s Disease Dementia
AUC	Area under the receiver operating characteristic curve
BBMs	Blood-Based Markers
CI	Confidence Interval
CDR	Clinical Dementia Rating
CSF	Cerebrospinal Fluid
DSM	Diagnostic and Statistical Manual of Mental Disorders
ELISA	Enzyme-linked immunosorbent assay
FU	Follow-up
MCI	Mild Cognitive Impairment
MMSE	Mini-Mental State Examination
MSA	Mass Spectrometry Assays
MSD	Meso Scale Discovery
NDDs	Neurodegenerative Diseases
NIA-AA	National Institute on Aging and Alzheimer’s Association
NINCDS-ADRDA	National Institute of Neurological and Communicative Disorders and Stroke and the Alzheimer’s Disease and Related Disorders Association
PET	Positron Emission Tomography
QUADAS-2	Quality Assessment of Diagnostic Accuracy Studies-2 version
SCD	Subjective Cognitive Decline
Simoa	Single-molecule array
SMD	Standardized Mean Difference

## References

[1] Beach T.G., Monsell S.E., Phillips L.E., Kukull W. (2012). Accuracy of the clinical diagnosis of Alzheimer disease at National Institute on Aging Alzheimer Disease Centers, 2005–2010. J. Neuropathol. Exp. Neurol..

[2] Souchet B., Michaïl A., Heuillet M., Dupuy-Gayral A., Haudebourg E., Pech C., Berthemy A.A., Autelitano F., Billoir B., Domoto-Reilly K. (2024). Multiomics Blood-Based Biomarkers Predict Alzheimer’s Predementia with High Specificity in a Multicentric Cohort Study. J. Prev. Alzheimers Dis..

[3] Dubois B., Feldman H.H., Jacova C., Dekosky S.T., Barberger-Gateau P., Cummings J., Delacourte A., Galasko D., Gauthier S., Jicha G. (2007). Research criteria for the diagnosis of Alzheimer’s disease: Revising the NINCDS-ADRDA criteria. Lancet Neurol..

[4] Jack C.R., Bennett D.A., Blennow K., Carrillo M.C., Dunn B., Haeberlein S.B., Holtzman D.M., Jagust W., Jessen F., Karlawish J. (2018). NIA-AA Research Framework: Toward a biological definition of Alzheimer’s disease. Alzheimers Dement..

[5] Jack C.R., Andrews J.S., Beach T.G., Buracchio T., Dunn B., Graf A., Hansson O., Ho C., Jagust W., McDade E. (2024). Revised criteria for diagnosis and staging of Alzheimer’s disease: Alzheimer’s Association Workgroup. Alzheimers Dement..

[6] Palmqvist S., Tideman P., Mattsson-Carlgren N., Schindler S.E., Smith R., Ossenkoppele R., Calling S., West T., Monane M., Verghese P.B. (2024). Blood Biomarkers to Detect Alzheimer Disease in Primary Care and Secondary Care. JAMA.

[7] Janelidze S., Barthélemy N.R., Salvadó G., Schindler S.E., Palmqvist S., Mattsson-Carlgren N., Braunstein J.B., Ovod V., Bollinger J.G., He Y. (2024). Plasma Phosphorylated Tau 217 and Aβ42/40 to Predict Early Brain Aβ Accumulation in People Without Cognitive Impairment. JAMA Neurol..

[8] Angioni D., Hansson O., Bateman R.J., Rabe C., Toloue M., Braunstein J.B., Agus S., Sims J.R., Bittner T., Carrillo M.C. (2023). Can We Use Blood Biomarkers as Entry Criteria and for Monitoring Drug Treatment Effects in Clinical Trials? A Report from the EU/US CTAD Task Force. J. Prev. Alzheimers Dis..

[9] Hansson O., Edelmayer R.M., Boxer A.L., Carrillo M.C., Mielke M.M., Rabinovici G.D., Salloway S., Sperling R., Zetterberg H., Teunissen C.E. (2022). The Alzheimer’s Association appropriate use recommendations for blood biomarkers in Alzheimer’s disease. Alzheimers Dement..

[10] Janelidze S., Mattsson N., Palmqvist S., Smith R., Beach T.G., Serrano G.E., Chai X., Proctor N.K., Eichenlaub U., Zetterberg H. (2020). Plasma P-tau181 in Alzheimer’s disease: Relationship to other biomarkers, differential diagnosis, neuropathology and longitudinal progression to Alzheimer’s dementia. Nat. Med..

[11] Ashton N.J., Pascoal T.A., Karikari T.K., Benedet A.L., Lantero-Rodriguez J., Brinkmalm G., Snellman A., Schöll M., Troakes C., Hye A. (2021). Plasma p-tau231: A new biomarker for incipient Alzheimer’s disease pathology. Acta Neuropathol..

[12] Palmqvist S., Janelidze S., Quiroz Y.T., Zetterberg H., Lopera F., Stomrud E., Su Y., Chen Y., Serrano G.E., Leuzy A. (2020). Discriminative Accuracy of Plasma Phospho-tau217 for Alzheimer Disease vs Other Neurodegenerative Disorders. JAMA.

[13] Therriault J., Vermeiren M., Servaes S., Tissot C., Ashton N.J., Benedet A.L., Karikari T.K., Lantero-Rodriguez J., Brum W.S., Lussier F.Z. (2023). Association of Phosphorylated Tau Biomarkers With Amyloid Positron Emission Tomography vs Tau Positron Emission Tomography. JAMA Neurol..

[14] Barthélemy N.R., Horie K., Sato C., Bateman R.J. (2020). Blood Plasma Phosphorylated-Tau Isoforms Track CNS Change in Alzheimer’s Disease. J. Exp. Med..

[15] Antonioni A., Raho E.M., Di Lorenzo F. (2024). Is blood pTau a reliable indicator of the CSF status? A narrative review. Neurol. Sci..

[16] Kac P.R., Gonzalez-Ortiz F., Simrén J., Dewit N., Vanmechelen E., Zetterberg H., Blennow K., Ashton N.J., Karikari T.K. (2022). Diagnostic value of serum versus plasma phospho-tau for Alzheimer’s disease. Alzheimers Res. Ther..

[17] Bayoumy S., Verberk I.M.W., den Dulk B., Hussainali Z., Zwan M., van der Flier W.M., Ashton N.J., Zetterberg H., Blennow K., Vanbrabant J. (2021). Clinical and analytical comparison of six Simoa assays for plasma P-tau isoforms P-tau181, P-tau217, and P-tau231. Alzheimers Res. Ther..

[18] Mielke M.M., Dage J.L., Frank R.D., Algeciras-Schimnich A., Knopman D.S., Lowe V.J., Bu G., Vemuri P., Graff-Radford J., Jack C.R. (2022). Performance of plasma phosphorylated tau 181 and 217 in the community. Nat. Med..

[19] Rissin D.M., Kan C.W., Campbell T.G., Howes S.C., Fournier D.R., Song L., Piech T., Patel P.P., Chang L., Rivnak A.J. (2010). Single-molecule enzyme-linked immunosorbent assay detects serum proteins at subfemtomolar concentrations. Nat. Biotechnol..

[20] O’Bryant S.E., Xiao G., Zhang F., Edwards M., German D.C., Yin X., Como T., Reisch J., Huebinger R.M., Graff-Radford N. (2014). Validation of a serum screen for Alzheimer’s disease across assay platforms, species, and tissues. J. Alzheimer’s Dis..

[21] Sato C., Barthélemy N.R., Mawuenyega K.G., Patterson B.W., Gordon B.A., Jockel-Balsarotti J., Sullivan M., Crisp M.J., Kasten T., Kirmess K.M. (2018). Tau Kinetics in Neurons and the Human Central Nervous System. Neuron.

[22] Schindler S.E., Galasko D., Pereira A.C., Rabinovici G.D., Salloway S., Suárez-Calvet M., Khachaturian A.S., Mielke M.M., Udeh-Momoh C., Weiss J. (2024). Acceptable performance of blood biomarker tests of amyloid pathology—Recommendations from the Global CEO Initiative on Alzheimer’s Disease. Nat. Rev. Neurol..

[23] Karikari T.K., Pascoal T.A., Ashton N.J., Janelidze S., Benedet A.L., Rodriguez J.L., Chamoun M., Savard M., Kang M.S., Therriault J. (2020). Blood phosphorylated tau 181 as a biomarker for Alzheimer’s disease: A diagnostic performance and prediction modelling study using data from four prospective cohorts. Lancet Neurol..

[24] Thijssen E.H., La Joie R., Strom A., Fonseca C., Iaccarino L., Wolf A., Spina S., Allen I.E., Cobigo Y., Heuer H. (2021). Plasma phosphorylated tau 217 and phosphorylated tau 181 as biomarkers in Alzheimer’s disease and frontotemporal lobar degeneration: A retrospective diagnostic performance study. Lancet Neurol..

[25] Ashton N.J., Puig-Pijoan A., Milà-Alomà M., Fernández-Lebrero A., García-Escobar G., González-Ortiz F., Kac P.R., Brum W.S., Benedet A.L., Lantero-Rodriguez J. (2023). Plasma and CSF biomarkers in a memory clinic: Head-to-head comparison of phosphorylated tau immunoassays. Alzheimers Dement..

[26] Therriault J., Servaes S., Tissot C., Rahmouni N., Ashton N.J., Benedet A.L., Karikari T.K., Macedo A.C., Lussier F.Z., Stevenson J. (2023). Equivalence of plasma p-tau217 with cerebrospinal fluid in the diagnosis of Alzheimer’s disease. Alzheimers Dement..

[27] Barthélemy N.R., Salvadó G., Schindler S.E., He Y., Janelidze S., Collij L.E., Saef B., Henson R.L., Chen C.D., Gordon B.A. (2024). Highly accurate blood test for Alzheimer’s disease is similar or superior to clinical cerebrospinal fluid tests. Nat. Med..

[28] Ashton N.J., Brum W.S., Di Molfetta G., Benedet A.L., Arslan B., Jonaitis E., Langhough R.E., Cody K., Wilson R., Carlsson C.M. (2024). Diagnostic Accuracy of a Plasma Phosphorylated Tau 217 Immunoassay for Alzheimer Disease Pathology. JAMA Neurol..

[29] Palmqvist S., Tideman P., Cullen N., Zetterberg H., Blennow K., Dage J.L., Stomrud E., Janelidze S., Mattsson-Carlgren N., Hansson O. (2021). Prediction of Future Alzheimer’s Disease Dementia Using Plasma Phospho-Tau Combined with Other Accessible Measures. Nat. Med..

[30] Lehmann S., Schraen-Maschke S., Vidal J.S., Delaby C., Buee L., Blanc F., Paquet C., Allinquant B., Bombois S., Gabelle A. (2024). Clinical value of plasma ALZpath pTau217 immunoassay for assessing mild cognitive impairment. J. Neurol. Neurosurg. Psychiatry.

[31] Janelidze S., Bali D., Ashton N.J., Barthélemy N.R., Vanbrabant J., Stoops E., Vanmechelen E., He Y., Dolado A.O., Triana-Baltzer G. (2023). Head-to-head comparison of 10 plasma phospho-tau assays in prodromal Alzheimer’s disease. Brain.

[32] Ashton N.J., Janelidze S., Mattsson-Carlgren N., Binette A.P., Strandberg O., Brum W.S., Karikari T.K., González-Ortiz F., Di Molfetta G., Meda F.J. (2022). Differential roles of Aβ42/40, p-tau231 and p-tau217 for Alzheimer’s trial selection and disease monitoring. Nat. Med..

[33] Du L., Langhough R.E., Wilson R.E., Reyes R.E.R., Hermann B.P., Jonaitis E.M., Betthauser T.J., Chin N.A., Christian B., Chaby L. (2024). Longitudinal plasma phosphorylated-tau217 and other related biomarkers in a non-demented Alzheimer’s risk-enhanced sample. Alzheimers Dement..

[34] Milà-Alomà M., Ashton N.J., Shekari M., Salvadó G., Ortiz-Romero P., Montoliu-Gaya L., Benedet A.L., Karikari T.K., Lantero-Rodriguez J., Vanmechelen E. (2022). Plasma P-Tau231 and p-Tau217 as State Markers of Amyloid-β Pathology in Preclinical Alzheimer’s Disease. Nat. Med..

[35] Mattsson-Carlgren N., Janelidze S., Palmqvist S., Cullen N., Svenningsson A.L., Strandberg O., Mengel D., Walsh D.M., Stomrud E., Dage J.L. (2020). Longitudinal plasma p-tau217 is increased in early stages of Alzheimer’s disease. Brain.

[36] Janelidze S., Palmqvist S., Leuzy A., Stomrud E., Verberk I.M.W., Zetterberg H., Ashton N.J., Pesini P., Sarasa L., Allué J.A. (2022). Detecting amyloid positivity in early Alzheimer’s disease using combinations of plasma Aβ42/Aβ40 and p-tau. Alzheimers Dement..

[37] Janelidze S., Berron D., Smith R., Strandberg O., Proctor N.K., Dage J.L., Stomrud E., Palmqvist S., Mattsson-Carlgren N., Hansson O. (2021). Associations of Plasma Phospho-Tau217 Levels With Tau Positron Emission Tomography in Early Alzheimer Disease. JAMA Neurol..

[38] Martínez-Dubarbie F., López-García S., Lage C., Di Molfetta G., Fernández-Matarrubia M., Pozueta-Cantudo A., García-Martínez M., Corrales-Pardo A., Bravo M., Jiménez-Bonilla J. (2024). Plasma Phosphorylated Tau 231 Increases at One-Year Intervals in Cognitively Unimpaired Subjects. J. Alzheimers Dis..

[39] Li H.-X., Wang J.-T., Dong Y., Li J.-P., Xiao J.-W., Ren R.-J., Li C.-B., Wang G. (2023). Blood Biomarkers in MCI Conversion to Alzheimer’s Disease: A Systematic Review and Meta-Analysis. Hum. Brain.

[40] Page M.J., McKenzie J.E., Bossuyt P.M., Boutron I., Hoffmann T.C., Mulrow C.D., Shamseer L., Tetzlaff J.M., Akl E.A., Brennan S.E. (2021). The PRISMA 2020 statement: An updated guideline for reporting systematic reviews. BMJ.

[41] Dubois B., Hampel H., Feldman H.H., Scheltens P., Aisen P., Andrieu S., Bakardjian H., Benali H., Bertram L., Blennow K. (2016). Proceedings of the Meeting of the International Working Group (IWG) and the American Alzheimer’s Association on “The Preclinical State of AD”; July 23, 2015; Washington DC, USA. Preclinical Alzheimer’s disease: Definition, natural history, and diagnostic criteria. Alzheimers Dement..

[42] Albert M.S., DeKosky S.T., Dickson D., Dubois B., Feldman H.H., Fox N.C., Gamst A., Holtzman D.M., Jagust W.J., Petersen R.C. (2011). The diagnosis of mild cognitive impairment due to Alzheimer’s disease: Recommendations from the National Institute on Aging-Alzheimer’s Association workgroups on diagnostic guidelines for Alzheimer’s disease. Alzheimers Dement..

[43] Petersen R.C., Smith G.E., Waring S.C., Ivnik R.J., Tangalos E.G., Kokmen E. (1999). Mild cognitive impairment: Clinical characterization and outcome. Arch. Neurol..

[44] Petersen R.C. (2004). Mild cognitive impairment as a diagnostic entity. J. Intern. Med..

[45] Winblad B., Palmer K., Kivipelto M., Jelic V., Fratiglioni L., Wahlund L.O., Nordberg A., Bäckman L., Albert M., Almkvist O. (2004). Mild cognitive impairment--beyond controversies, towards a consensus: Report of the International Working Group on Mild Cognitive Impairment. J. Intern. Med..

[46] Sachdev P.S., Blacker D., Blazer D.G., Ganguli M., Jeste D.V., Paulsen J.S., Petersen R.C. (2014). Classifying neurocognitive disorders: The DSM-5 approach. Nat. Rev. Neurol..

[47] Morris J.C. (1993). The Clinical Dementia Rating (CDR): Current version and scoring rules. Neurology.

[48] McKhann G., Drachman D., Folstein M., Katzman R., Price D., Stadlan E.M. (1984). Clinical diagnosis of Alzheimer’s disease: Report of the NINCDS-ADRDA Work Group under the auspices of Department of Health and Human Services Task Force on Alzheimer’s Disease. Neurology.

[49] McKhann G.M., Knopman D.S., Chertkow H., Hyman B.T., Jack C.R., Kawas C.H., Klunk W.E., Koroshetz W.J., Manly J.J., Mayeux R. (2011). The diagnosis of dementia due to Alzheimer’s disease: Recommendations from the National Institute on Aging-Alzheimer’s Association workgroups on diagnostic guidelines for Alzheimer’s disease. Alzheimers Dement..

[50] Dubois B., Feldman H.H., Jacova C., Hampel H., Molinuevo J.L., Blennow K., DeKosky S.T., Gauthier S., Selkoe D., Bateman R. (2014). Advancing research diagnostic criteria for Alzheimer’s disease: The IWG-2 criteria. Lancet Neurol..

[51] Whiting P.F., Rutjes A.W., Westwood M.E., Mallett S., Deeks J.J., Reitsma J.B., Leeflang M.M., Sterne J.A., Bossuyt P.M., QUADAS-2 Group (2011). QUADAS-2: A revised tool for the quality assessment of diagnostic accuracy studies. Ann. Intern. Med..

[52] Andrade C. (2020). Mean Difference, Standardized Mean Difference (SMD), and Their Use in Meta-Analysis: As Simple as It Gets. J. Clin. Psychiatry..

[53] Salgado J.F. (2018). Transforming the area under the normal curve (AUC) into Cohen’sd, Pearson’s rpb, odds-ratio, and natural log odds-ratio: Two conversion tables. Eur. J. Psychol. Appl. Leg. Context..

[54] de Hond A.A.H., Steyerberg E.W., van Calster B. (2022). Interpreting area under the receiver operating characteristic curve. Lancet Digit. Health.

[55] Higgins J. (2003). Measuring inconsistency in meta-analyses. BMJ.

[56] Cai Y., Fan X., Zhao L., Liu W., Luo Y., Lau A.Y.L., Au L.W.C., Shi L., Lam B.Y.K., Ko H. (2023). Comparing machine learning-derived MRI-based and blood-based neurodegeneration biomarkers in predicting syndromal conversion in early AD. Alzheimers Dement..

[57] Kwon H.S., Kim J.Y., Koh S.H., Choi S.H., Lee E.H., Jeong J.H., Jang J.W., Park K.W., Kim E.J., Hong J.Y. (2023). Predicting cognitive stage transition using p-tau181, Centiloid, and other measures. Alzheimers Dement..

[58] Pichet Binette A., Palmqvist S., Bali D., Farrar G., Buckley C.J., Wolk D.A., Zetterberg H., Blennow K., Janelidze S., Hansson O. (2022). Combining plasma phospho-tau and accessible measures to evaluate progression to Alzheimer’s dementia in mild cognitive impairment patients. Alzheimers Res. Ther..

[59] Palmqvist S., Stomrud E., Cullen N., Janelidze S., Manuilova E., Jethwa A., Bittner T., Eichenlaub U., Suridjan I., Kollmorgen G. (2023). An accurate fully automated panel of plasma biomarkers for Alzheimer’s disease. Alzheimers Dement..

[60] Planche V., Bouteloup V., Pellegrin I., Mangin J.-F., Dubois B., Ousset P.-J., Pasquier F., Blanc F., Paquet C., Hanon O. (2023). Validity and Performance of Blood Biomarkers for Alzheimer Disease to Predict Dementia Risk in a Large Clinic-Based Cohort. Neurology.

[61] Chen T.B., Lai Y.H., Ke T.L., Chen J.P., Lee Y.J., Lin S.Y., Lin P.C., Wang P.N., Cheng I.H. (2019). Changes in Plasma Amyloid and Tau in a Longitudinal Study of Normal Aging, Mild Cognitive Impairment, and Alzheimer’s Disease. Dement. Geriatr. Cogn. Disord..

[62] Kivisäkk P., Carlyle B.C., Sweeney T., Trombetta B.A., LaCasse K., El-Mufti L., Tuncali I., Chibnik L.B., Das S., Scherzer C.R. (2023). Plasma biomarkers for diagnosis of Alzheimer’s disease and prediction of cognitive decline in individuals with mild cognitive impairment. Front. Neurol..

[63] Silva-Spinola A., Lima M., Leitão M.J., Bernardes C., Durães J., Duro D., Tábuas-Pereira M., Santana I., Baldeiras I. (2023). Blood biomarkers in mild cognitive impairment patients: Relationship between analytes and progression to Alzheimer disease dementia. Eur. J. Neurol..

[64] Simrén J., Leuzy A., Karikari T.K., Hye A., Benedet A.L., Lantero-Rodriguez J., Mattsson-Carlgren N., Schöll M., Mecocci P., Vellas B. (2021). The diagnostic and prognostic capabilities of plasma biomarkers in Alzheimer’s disease. Alzheimers Dement..

[65] Yuan M., Lian S., Li X., Long X., Fang Y., Alzheimer’s Disease Neuroimaging Initiative (ADNI) (2024). Blood biomarkers in dynamic prediction of conversion to Alzheimer’s disease: An application of joint modeling. Int. J. Geriatr. Psychiatry..

[66] Park M.K., Ahn J., Kim Y.J., Lee J.W., Lee J.C., Hwang S.J., Kim K.C. (2024). Predicting Longitudinal Cognitive Decline and Alzheimer’s Conversion in Mild Cognitive Impairment Patients Based on Plasma Biomarkers. Cells.

[67] Sanchez E., Wilkinson T., Coughlan G., Mirza S., Baril A.A., Ramirez J., Binns M.A., Black S.E., Borrie M., Dilliott A.A. (2024). Association of plasma biomarkers with cognition, cognitive decline, and daily function across and within neurodegenerative diseases: Results from the Ontario Neurodegenerative Disease Research Initiative. Alzheimers Dement..

[68] Martínez G., Vernooij R.W., Fuentes Padilla P., Zamora J., Bonfill Cosp X., Flicker L. (2017). 18F PET with florbetapir for the early diagnosis of Alzheimer’s disease dementia and other dementias in people with mild cognitive impairment (MCI). Cochrane Database Syst Rev..

[69] Cullen N.C., Leuzy A., Palmqvist S., Janelidze S., Stomrud E., Pesini P., Sarasa L., Allué J.A., Proctor N.K., Zetterberg H. (2021). Individualized prognosis of cognitive decline and dementia in mild cognitive impairment based on plasma biomarker combinations. Nat. Aging.

[70] Karikari T.K., Benedet A.L., Ashton N.J., Lantero Rodriguez J., Snellman A., Suárez-Calvet M., Saha-Chaudhuri P., Lussier F., Kvartsberg H., Rial A.M. (2021). Diagnostic performance and prediction of clinical progression of plasma phospho-tau181 in the Alzheimer’s Disease Neuroimaging Initiative. Mol. Psychiatry.

[71] Shen X.N., Huang Y.Y., Chen S.D., Guo Y., Tan L., Dong Q., Yu J.T., Alzheimer’s Disease Neuroimaging Initiative (2021). Plasma phosphorylated-tau181 as a predictive biomarker for Alzheimer’s amyloid, tau and FDG PET status. Transl. Psychiatry.

[72] Therriault J., Benedet A.L., Pascoal T.A., Lussier F.Z., Tissot C., Karikari T.K., Ashton N.J., Chamoun M., Bezgin G., Mathotaarachchi S. (2021). Alzheimer’s Disease Neuroimaging Initiative. Association of plasma P-tau181 with memory decline in non-demented adults. Brain Commun..

[73] Kivisäkk P., Magdamo C., Trombetta B.A., Noori A., Kuo Y.K.E., Chibnik L.B., Carlyle B.C., Serrano-Pozo A., Scherzer C.R., Hyman B.T. (2022). Plasma biomarkers for prognosis of cognitive decline in patients with mild cognitive impairment. Brain Commun..

[74] Xiao Z., Wu W., Ma X., Liang X., Lu J., Zheng L., Ding S., Lei Q., Luo J., Chen K. (2022). Plasma Aβ42/Aβ40 and p-tau181 Predict Long-Term Clinical Progression in a Cohort with Amnestic Mild Cognitive Impairment. Clin. Chem..

[75] Lehmann S., Schraen-Maschke S., Vidal J.S., Delaby C., Blanc F., Paquet C., Allinquant B., Bombois S., Gabelle A., Hanon O. (2023). Plasma phosphorylated tau 181 predicts amyloid status and conversion to dementia stage dependent on renal function. J. Neurol. Neurosurg. Psychiatry.

[76] Ma Y., Brettschneider J., Collingwood J.F. (2022). A Systematic Review and Meta-Analysis of Cerebrospinal Fluid Amyloid and Tau Levels Identifies Mild Cognitive Impairment Patients Progressing to Alzheimer’s Disease. Biomedicines.

[77] Salvadó G., Larsson V., Cody K.A., Cullen N.C., Jonaitis E.M., Stomrud E., Kollmorgen G., Wild N., Palmqvist S., Janelidze S. (2023). Optimal combinations of CSF biomarkers for predicting cognitive decline and clinical conversion in cognitively unimpaired participants and mild cognitive impairment patients: A multi-cohort study. Alzheimers Dement..

[78] Schöll M., Verberk I.M.W., Del Campo M., Delaby C., Therriault J., Chong J.R., Palmqvist S., Alcolea D. (2024). Challenges in the practical implementation of blood biomarkers for Alzheimer’s disease. Lancet Healthy Longev..

[79] Mattke S., Jun H., Hanson M., Chu S., Kordower J.H., Reiman E.M. (2024). Health Economic Considerations in the Deployment of an Alzheimer’s Prevention Therapy. J. Prev. Alzheimers Dis..

[80] Angioni D., Delrieu J., Hansson O., Fillit H., Aisen P., Cummings J., Sims J.R., Braunstein J.B., Sabbagh M., Bittner T. (2022). Blood Biomarkers from Research Use to Clinical Practice: What Must Be Done? A Report from the EU/US CTAD Task Force. J. Prev. Alzheimers Dis..

[81] Tsiknia A.A., Edland S.D., Sundermann E.E., Reas E.T., Brewer J.B., Galasko D., Banks S.J., Alzheimer’s Disease Neuroimaging Initiative (2022). Sex differences in plasma p-tau181 associations with Alzheimer’s disease biomarkers, cognitive decline, and clinical progression. Mol. Psychiatry..

[82] Bouteloup V., Pellegrin I., Dubois B., Chene G., Planche V., Dufouil C., MEMENTO Study Group (2024). Explaining the Variability of Alzheimer Disease Fluid Biomarker Concentrations in Memory Clinic Patients Without Dementia. Neurology.

[83] Dhauria M., Mondal R., Deb S., Shome G., Chowdhury D., Sarkar S., Benito-León J. (2024). Blood-Based Biomarkers in Alzheimer’s Disease: Advancing Non-Invasive Diagnostics and Prognostics. Int. J. Mol. Sci..

[84] Gonzalez-Ortiz F., Kac P.R., Brum W.S., Zetterberg H., Blennow K., Karikari T.K. (2023). Plasma phospho-tau in Alzheimer’s disease: Towards diagnostic and therapeutic trial applications. Mol. Neurodegener..

[85] Moscoso A., Grothe M.J., Ashton N.J., Karikari T.K., Rodriguez J.L., Snellman A., Suárez-Calvet M., Zetterberg H., Blennow K., Alzheimer’s Disease Neuroimaging Initiative (2021). Time course of phosphorylated-tau181 in blood across the Alzheimer’s disease spectrum. Brain.

[86] Bellomo G., Bayoumy S., Megaro A., Toja A., Nardi G., Gaetani L., Blujdea E.R., Paolini Paoletti F., Wojdaƚa A.L., Chiasserini D. (2024). Fully automated measurement of plasma Aβ42/40 and p-tau181: Analytical robustness and concordance with cerebrospinal fluid profile along the Alzheimer’s disease continuum in two independent cohorts. Alzheimers Dement..

