# Supplementary material for: Role of Blood P-Tau Isoforms (181, 217, 231) in Predicting Conversion from MCI to Dementia Due to Alzheimer’s Disease: A Review and Meta-Analysis
Source: Int J Mol Sci. 2024 Nov 30;25(23):12916. doi: 10.3390/ijms252312916 (PMC11641364; doi:10.3390/ijms252312916)
Supplement: Supplementary file 1 [file ijms-25-12916-s001.zip › Supplementary Table S1_ptau isoforms.pdf]

Supplementary Table S1: Shared (fist column) and peculiar (other columns) characteristics of p-tau isoforms. AD: Alzheimer's Disease; ADD: Alzheimer's Disease Dementia (ADD); CSF: cerebrospinal fluid; CU: Cognitive Unimpaired; MCI: Mild Cognitive Impairment

**BLOOD P-TAU ISOFORM SHARED CHARACTERISTICS**	**BLOOD P-TAU ISOFORM PECULIAR CHARACTERISTICS**	TIMING OF INCREASE	CORRELATION WITH PET IMAGING	CORRELATION WITH CSF BIOMARKERS	CLINICAL UTILITY
p-tau shows higher specificity for AD, indeed an increase in this BBM has been observed only in AD and not in other tauopathies [9]<br><br>p-tau have significantly higher correlation with Amyloid PET than with Tau PET [13]<br><br>In case of detecting symptomatic AD, all p-tau isoforms perform equally well [84]<br><br>Significant correlation between isoforms p-tau in blood and CSF [15]<br><br>The AUC for conversion to AD in	**p-tau 181**	p-tau 181 reached abnormal levels significantly later (6 years) than CSF amyloid β1–42 and Amyloid PET, following similar dynamics of CSF p-tau181 [85]<br><br>p-tau 181 is the last p-tau that starts to change [15]	Voxel-wise PET analyses yielded relatively weak, yet significant, associations with amyloid-β pathology in CU, while the strongest associations with amyloid-β were observed in late accumulating regions in patients with MCI. Cross-sectional and particularly longitudinal measures of plasma p-tau181 were associated with widespread cortical tau aggregation 6 years later [85]	p-tau 181 levels accurately reflected CSF A+/T+ profile in ADD and (MCI)-AD, but not in asymptomatic-AD [86]	Prediction to conversion to ADD in MCI [10,23] with similar accuracy vs CSF [70]<br><br>Differential diagnosis between AD e non-AD pathology [10,23,24,70]

MCI is significant for both p-tau 181 and 217 but lower than that is for the detection of cerebral amyloidosis [30]					
	**p-tau 217**	It starts to increase in CU individuals [34,35,36]<br><br>It increases in the prodromal stage of AD [35]	It can predict cerebral amyloid pathology assessed by Amyloid PET [36]<br><br>It start becoming abnormal before Tau PET [37] (<br><br>p-tau 217 trajectory is different in Amyloid PET positive vs Amyloid PET negative subjects: average p-tau 217 change rates is higher in subjects who are amyloid positive [33]<br><br>p-tau 217 reaches abnormal levels at 35.4 Centiloids [34]	p-tau 217 show strongest association with amyloid pathology in MCI but not in CU [36]	Prediction conversion to ADD in MCI, similar accuracy vs CSF [29] , superior to plasma p-tau 181 [30,31]<br><br>Longitudinal increase in p-tau 217 is a marker of disease progression in preclinical and prodromal AD [28,32]<br><br>Higher plasma p-tau 217 is associated with faster preclinical cognitive decline [33]<br><br>Combining plasma p-tau 217and Aβ42/40 levels could be useful for predicting development of Aβ pathology in people with early stages of subthreshold Aβ accumulation, facilitate screening of participants for future primary prevention trials [7]<br><br>The amyloid probability score 2 [APS2] obtained combining blood p-tau 217 with Aβ42/40 markers or percentage of p-tau 217 alone accurately identify AD in primary and secondary care providing superior performance in comparison with the diagnostic accuracy obtained using standard

					clinical evaluation in patients with cognitive symptoms [6]<br><br>Differential diagnosis between AD e non-AD [12]; similar accuracy vs CSF [12,26,27,28], similar [25], or superior to plasma p-tau 181 [12,24]
	**p-tau 231**	It is the earlier to change [11,15,34]	It might be altered before Amyloid PET positivity and in response to early brain Tau deposition [11]<br><br>p-tau 231 reaches abnormal levels at 26,4 Centiloids [34]	In Cognive Unimpaired, plasma p-tau 231 rises as Aβ42/40 drops in CSF [34]	p-tau231 is able to distinguish CU Aβ-positive participants from Aβ-negative MCI cases with higher accuracy and significantly superior to plasma p-tau181<br>It could be used to detect population susceptible to AD in CU [11]<br><br>Basal levels of p-tau231 correlate negatively with memory tests only in subjects with a sufficient amyloid load [38]<br><br>Differential diagnosis between AD e non-AD [11]
