# Supplementary material for: Role of Blood P-Tau Isoforms (181, 217, 231) in Predicting Conversion from MCI to Dementia Due to Alzheimer’s Disease: A Review and Meta-Analysis
Source: Int J Mol Sci. 2024 Nov 30;25(23):12916. doi: 10.3390/ijms252312916 (PMC11641364; doi:10.3390/ijms252312916)
Supplement: Supplementary file 1 [file ijms-25-12916-s001.zip › Supplementary Table S2-S4.pdf]

Supplementary table S2: Embase search string

No.	Query	Results
#8	#5 NOT #6 AND [english]/lim	677
#7	#5 NOT #6	683
#6	(#1 OR #2) AND ([article]/lim OR [article in press]/lim) NOT ('cross-sectional study' OR 'case report' OR 'case study') AND ([animal cell]/lim OR [animal experiment]/lim OR [animal model]/lim OR [animal tissue]/lim)	15
#5	(#1 OR #2) AND ([article]/lim OR [article in press]/lim) NOT ('cross-sectional study' OR 'case report' OR 'case study')	698
#4	(#1 OR #2) AND ([article]/lim OR [article in press]/lim)	832
#3	#1 OR #2	1433
#2	('mild cognitive impairment'/syn OR 'preclinical alzheimer s disease' OR 'preclinical ad' OR 'prodromal alzheimer s disease' OR 'prodromal ad' OR 'early alzheimer s disease' OR 'early ad' OR 'minor neurocognitive disorder' OR 'minor neurocognitive disorders' OR 'cognitive impairment no dementia' OR cind) AND ('alzheimer disease'/syn OR 'dementia'/syn) AND ('blood biomarker' OR 'blood biomarkers' OR 'serum biomarker' OR 'serum biomarkers' OR 'plasma biomarker' OR 'plasma biomarkers' OR 'blood biological markers' OR 'blood biological marker' OR 'plasma biological marker' OR 'plasma biological markers' OR 'serum biological marker' OR 'serum biological markers' OR 'phospho tau' OR 'phosphorylated tau' OR 'p-tau isoform' OR 'p-tau isoforms' OR 'ptau isoform' OR 'ptau isoforms' OR 'p tau isoform' OR 'p tau isoforms' OR ptau217 OR ptau231 OR 'p tau181' OR 'p tau217' OR 'p tau231') AND ('prediction'/syn OR progress* OR conver* OR 'prognosis'/syn OR 'longitudinal study'/syn OR 'follow up'/syn)	1226
#1	(mci OR 'mild cognitive impairment'/exp OR 'mild cognitive impairment' OR 'preclinical alzheimer s disease' OR 'preclinical ad' OR 'prodromal alzheimer s disease' OR 'prodromal ad' OR 'early alzheimer s disease' OR 'early ad' OR 'minor neurocognitive disorder' OR 'minor cognitive disorders' OR 'cognitive impairment no dementia' OR cind) AND ('alzheimer s disease dementia' OR 'dementia'/exp OR dementia) AND ('blood biomarker' OR 'blood biomarkers' OR 'serum biomarker' OR 'serum biomarkers' OR 'plasma biomarker' OR 'plasma biomarkers' OR 'blood biological marker' OR 'blood biological markers' OR 'plasma biological marker' OR 'plasma biological markers' OR 'serum biological marker' OR 'serum biological markers' OR 'phospho tau' OR 'phosphorylated tau' OR 'p-tau isoform' OR 'p tau isoform' OR 'ptau isoforms' OR 'p tau isoforms' OR ptau181 OR ptau217 OR ptau231 OR 'p tau181' OR 'p tau217' OR 'p tau231') AND ('prediction'/exp OR prediction	1281

	OR progression OR conversion OR 'prognosis'/exp OR prognosis OR longitudinal OR 'follow up'/exp OR 'follow up')	

Supplementary table S3: Pubmed search string

No.	Query	Filters	Results
#20	#18 NOT #16	English	2077
#19	#18 NOT #16		2100
#18	(#2 NOT #4) NOT ("cross sectional stud*" OR "case stud*" OR "case serie*")		2623
#17	(#2 NOT #4) NOT ("cross sectional stud*" OR "case stud* OR "case serie*")		2913
#16	#2 NOT #4	Case Reports, Congress, Editorial, Guideline, Letter, Meta-Analysis, Practice Guideline, Preprint, Review, Systematic Review	551
#15	#2 NOT #4	Congress, Editorial, Guideline, Letter, Meta-Analysis, Practice Guideline, Preprint, Review, Systematic Review	533
#14	#2 NOT #4	Editorial, Guideline, Letter, Meta-Analysis, Practice Guideline, Preprint, Review, Systematic Review	530
#13	#2 NOT #4	Guideline, Letter, Meta-Analysis, Practice Guideline,	528

		Preprint, Review, Systematic Review	
#12	#2 NOT #4	Letter, Meta-Analysis, Practice Guideline, Preprint, Review, Systematic Review	528
#11	#2 NOT #4	Meta-Analysis, Practice Guideline, Preprint, Review, Systematic Review	521
#10	#2 NOT #4	Practice Guideline, Preprint, Review, Systematic Review	506
#9	#2 NOT #4	Preprint, Review, Systematic Review	505
#8	#2 NOT #4	Review, Systematic Review	485
#7	#2 NOT #4	Systematic Review	60
#6	#2 NOT #4		2918
#5	#2 NOT #4	Other Animals	0
#4	(MCI OR mild cognitive impairment OR Preclinical Alzheimer’s Disease OR "Preclinical AD" OR Prodromal Alzheimer’s Disease OR "Prodromal AD" OR early Alzheimer’s Disease OR "early AD" OR minor neurocognitive disorder OR "cognitive impairment no dementia" OR CIND) AND ("Alzheimer’s disease Dementia" OR Dementia) AND (blood biomarker OR serum biomarker OR plasma biomarker OR blood biological marker OR plasma biological marker OR serum biological marker OR "phospho tau" OR "phosphorylated tau" OR p-tau isoform OR ptau isoform OR ptau181 OR ptau217 OR ptau231 OR p-tau181 OR p-tau217 OR p-tau231) AND (prediction OR progression OR conversion OR prognosis OR longitudinal OR follow-up)	Other Animals	354

#3	(MCI OR mild cognitive impairment OR Preclinical Alzheimer's Disease OR "Preclinical AD" OR Prodromal Alzheimer's Disease OR "Prodromal AD" OR early Alzheimer's Disease OR "early AD" OR minor neurocognitive disorder OR "cognitive impairment no dementia" OR CIND) AND ("Alzheimer's disease Dementia" OR Dementia) AND (blood biomarker OR serum biomarker OR plasma biomarker OR blood biological marker OR plasma biological marker OR serum biological marker OR "phospho tau" OR "phosphorylated tau" OR p-tau isoform OR ptau isoform OR ptau181 OR ptau217 OR ptau231 OR p-tau181 OR p-tau217 OR p-tau231) AND (prediction OR progression OR conversion OR prognosis OR longitudinal OR follow-up)	Humans	2851
#2	(MCI OR mild cognitive impairment OR Preclinical Alzheimer's Disease OR "Preclinical AD" OR Prodromal Alzheimer's Disease OR "Prodromal AD" OR early Alzheimer's Disease OR "early AD" OR minor neurocognitive disorder OR "cognitive impairment no dementia" OR CIND) AND ("Alzheimer's disease Dementia" OR Dementia) AND (blood biomarker OR serum biomarker OR plasma biomarker OR blood biological marker OR plasma biological marker OR serum biological marker OR "phospho tau" OR "phosphorylated tau" OR p-tau isoform OR ptau isoform OR ptau181 OR ptau217 OR ptau231 OR p-tau181 OR p-tau217 OR p-tau231) AND (prediction OR progression OR conversion OR prognosis OR longitudinal OR follow-up)		3272
#1	(MCI OR mild cognitive impairment OR Preclinical Alzheimer's Disease OR "Preclinical AD" OR Prodromal Alzheimer's Disease OR "Prodromal AD" OR early Alzheimer's Disease OR "early AD" OR minor neurocognitive disorder OR "cognitive impairment no dementia" OR CIND) AND ("Alzheimer's disease Dementia" OR Dementia) AND (blood biomarker OR serum biomarker OR plasma biomarker OR blood biological marker OR plasma biological marker OR serum biological marker OR "phospho tau" OR "phosphorylated tau" OR p-tau isoform OR ptau isoform OR ptau181 OR ptau217 OR ptau231 OR p-tau181 OR p-tau217 OR p-tau231) AND (prediction OR progression OR conversion OR prognosis OR longitudinal OR follow-up)	English	3215

Supplementary table S4: Cochrane Library search string

No.	Query	Results
#23	#4 AND #8 AND #16 AND #22	408
#22	#17 OR #18 OR #19 OR #20 OR #21	677760
#21	MeSH descriptor: [Follow-Up Studies] explode all trees	75274
#20	MeSH descriptor: [Longitudinal Studies] explode all trees	8898
#19	MeSH descriptor: [Prognosis] explode all trees	228648
#18	MeSH descriptor: [Disease Progression] explode all trees	11084
#17	((predict* OR progress* OR convers* OR prognos* OR longitudinal OR follow-up)):ti,ab,kw	564074
#16	#9 OR #14 OR #15	42146
#15	('phospho tau' OR 'phosphorylated tau' OR 'p-tau isoform' OR 'p-tau isoforms' OR 'ptau isoform' OR 'ptau isoforms' OR 'p tau isoform' OR 'p tau isoforms' OR ptau217 OR ptau231 OR 'p tau181' OR 'p tau217' OR 'p tau231'):ti,ab,kw	313
#14	(#10 OR #11 OR #12) AND #13	2572
#13	MeSH descriptor: [Biomarkers] explode all trees	31951
#12	MeSH descriptor: [Plasma] explode all trees	2027
#11	MeSH descriptor: [Serum] explode all trees	1079
#10	MeSH descriptor: [Blood] explode all trees	21379
#9	(('blood biomarker' OR 'blood biomarkers' OR 'serum biomarker' OR 'serum biomarkers' OR 'plasma biomarker' OR 'plasma biomarkers' OR 'blood biological markers' OR 'blood biological marker' OR 'plasma biological marker' OR 'plasma biological markers' OR 'serum biological marker' OR 'serum biological markers' OR 'phospho tau' OR 'phosphorylated tau' OR 'p-tau isoform' OR 'p-tau isoforms' OR 'ptau isoform' OR 'ptau isoforms' OR 'p tau isoform' OR 'p tau isoforms' OR ptau217 OR ptau231 OR 'p tau181' OR 'p tau217' OR 'p tau231')):ti,ab,kw	40878
#8	#5 OR #6 OR #7	21667
#7	MeSH descriptor: [Dementia] explode all trees	9658
#6	MeSH descriptor: [Alzheimer Disease] explode all trees	5452
#5	(("Alzheimer’s disease Dementia" OR Dementia)):ti,ab,kw	18267
#4	#1 OR #2 OR #3	24556
#3	MeSH descriptor: [Neurocognitive Disorders] explode all trees	18431
#2	MeSH descriptor: [Cognitive Dysfunction] explode all trees	4205

#1	((MCI OR mild cognitive impairment OR Preclinical Alzheimer’s Disease OR "Preclinical AD" OR Prodromal Alzheimer’s Disease OR "Prodromal AD" OR early Alzheimer’s Disease OR "early AD" OR minor neurocognitive disorder OR "cognitive impairment no dementia" OR CIND)):ti,ab,kw	9456
