# Supplementary material for: Role of Blood P-Tau Isoforms (181, 217, 231) in Predicting Conversion from MCI to Dementia Due to Alzheimer’s Disease: A Review and Meta-Analysis
Source: Int J Mol Sci. 2024 Nov 30;25(23):12916. doi: 10.3390/ijms252312916 (PMC11641364; doi:10.3390/ijms252312916)
Supplement: Supplementary file 1 [file ijms-25-12916-s001.zip › Supplementary Table S5.pdf]

Review-specific QUADAS-2 scheme

**DOMAIN 1) PATIENT SELECTION**<br>**Quality assessment**	YES	NO	UNCLEAR	**REVIEWER JUDGEMENT ON RISK OF BIAS**<br>**(answer yes/no to the in bold question)**
Was a consecutive or random sample of patients enrolled?	Consecutive or random sampling of patients with MCI attending secondary or tertiary care	Opportunistic sampling based on volunteering or referral or selected case, including registries; retrospective studies with unclear sampling methods	Missing/ambiguous information on criteria	
Was a case-control design clearly avoided?	Prospective consecutive or random enrollment of patients with MCI	Patients with ADD and controls (without ADD) selected independently	Missing/ambiguous information	
Did the study avoid inappropriate exclusion?	Inclusion of cases with MCI seen at secondary care or memory clinics	Inappropriate exclusion of cases with comorbidities as depression, diabetes, cardiovascular disease or on MRI findings as vascular burden or other biomarker results	Missing/insufficient information	
**Risk of bias: Could the enrolment of participants have introduced bias? (selection bias, spectrum bias)**				Low: if “yes” answer for all of the 3 questions;<br>High: if “no” answer for any of the 3 questions;

				Unclear: if “unclear” answer for any of the 3 questions and an high risk was not applicable
**CONCERN REGARDING APPLICABILITY: Are there concerns that the included patients do not match the review question?**	Low: If the study include a clinically relevant population that are referred to secondary care or memory clinics by General Practitioners or other specialists and MCI is confirmed.<br>High: If the study population differed in terms of clinical features and comorbidity from that of interest for the review<br>Unclear: if this information is unclear			

**DOMAIN 2) INDEX TEST**<br>**Quality assessment**	YES	NO	UNCLEAR	**REVIEWER JUDGEMENT ON RISK OF BIAS**<br>**(answer yes/no to the in bold question)**
Were the index test results interpreted without knowledge of the results of the reference standard?	Index test assessed baseline in a prospective study (information on reference standard not available); or<br>Index test assessed retrospectively but it is clearly reported that data were collected in a prospective way and are referred to the baseline (implicit blindness of the laboratory staff)	Index test assessed retrospectively knowing the reference standard and/or blindness of the laboratory staff not reported	Missing/insufficient information	
If a threshold was used, was it pre-specified?	A pre-specified cut-off is available in the protocol or methods	A posteriori definition of a threshold (e.g. based on the Youden index) without validation in other cohorts	Missing/insufficient information	
Were the laboratory techniques used and laboratory staff appropriate?	Clearly reported and standardized procedures (e.g. SIMOA) used for all participants included in the study, trained laboratory	Not reported or not standardized or different laboratory methods used for the same cohort; laboratory	Missing/incomplete information; prototype assay/in-house method used	

	staff when manual assay used	staff not trained when manual assay used		
**Risk of bias: Could the conduct or interpretation of the index test have introduced bias? (test review bias, clinical review bias, methodology bias)**	Low: if “yes” answer for all of the 3 questions;<br>High: if “no” answer for any of the 3 questions;<br>Unclear: if “unclear” answer for any of the 3 questions and a high risk was not applicable or in case of “no” answer only for the second question			
**CONCERN REGARDING APPLICABILITY: Are there concerns that the index test, its conduct, or interpretation differ from the review question?**	Low: studies reporting p-tau values independently of the method used<br>Unclear: if repetitive measures with different methods were used to measure p-tau			

**DOMAIN 3) REFERENCE STANDARD Quality assessment**	YES	NO	UNCLEAR	**REVIEWER JUDGEMENT ON RISK OF BIAS (answer yes/no to the in bold question)**
Is the reference standard likely to correctly classify the target condition?	Diagnosis of ADD according to different clinical criteria (as NIA-AA criteria, IWG2) within the AD continuum (according to Jack 2018) with in vivo or post-mortem verification of the presence of A+ (tested by CSF or PET). Clinical diagnosis of other dementias according to clinical diagnostic criteria supported by biomarkers or neuropathology	Studies without clinical criteria reporting will be excluded according to exclusion criteria	Diagnosis of ADD without biomarkers/neuropathology confirmation	

Were the reference standard results interpreted without knowledge of the results of the index test?	Reference standard performed blinded or independently and without knowledge of index test	Reference standard performed not blinded (results of index test availability)	Unclear whether results were interpreted independently	
**Risk of bias: Could the reference standard, its conduct or its interpretation have introduced bias?**				Low: if “yes” answer for all of the 2 questions;<br>High: if “no” answer for any of the 2 questions<br>Unclear: if “unclear” classification for either of the 2 questions and “high risk” was not applicable
**CONCERN REGARDING APPLICABILITY: Are there concerns that the target condition as defined by the reference standard does not match the review question?**	Low: studies will be classified with low concern when accepted clinical criteria were used for the ADD diagnosis, and the diagnosis was biologically confirmed (A+)<br>Unclear: studied will be classified with unclear concern when only clinical criteria for the ADD diagnosis were used			

**DOMAIN 4) FLOW AND TIMING Quality assessment**	YES	NO	UNCLEAR	**REVIEWER JUDGEMENT ON RISK OF BIAS (answer yes/no to the in bold question)**
Was there an appropriate interval between index test and reference standard?	The time interval between index test and reference standard was at least 12 months for all participants; length of follow up clearly stated; a longer follow-up is accepted and clearly stated	A follow-up less than 12 months it is not permitted according to inclusion criteria (these studies will be excluded)	No details on the exact duration of follow-up (these studies will be excluded)	
Did all patients receive the same refence standard in the study?	All participants included in the study received the same reference standard; for	Participants of the same cohort received different reference standard	Missing/incomplete information	

	studies including different cohorts, clinical criteria used are reported as comparable in the inclusion criteria			
Were all patients included in the analysis?	Patients included in the study matched those in analyses	Patients included and analysed do not match: Reasons for withdrawals not explained; number of participants withdraw and lost not reported; exclusion of cases without a conversion from ADD.	Not clear whether all participants who entered the study, were accounted for or whether participants withdraw or lost in the follow-up differed from those who remained	
**Risk of bias: Could the patient flow have introduced bias?**				Low: if “yes” answer for all of the 3 questions;<br>High: if “no” or “unclear” answer in the third question<br>Unclear: if “unclear” answer for the first or the second question, and a high risk was not applicable
